# OIP5, a target of miR-15b-5p, regulates hepatocellular carcinoma growth and metastasis through the AKT/mTORC1 and β-catenin signaling pathways

**DOI:** 10.18632/oncotarget.15185

**Published:** 2017-02-08

**Authors:** Hua Li, Jun Zhang, Mi-Jin Lee, Goung-Ran Yu, Xueji Han, Dae-Ghon Kim

**Affiliations:** ^1^ Division of Gastroenterology and Hepatology, Department of Internal Medicine, The Research Institute of Clinical Medicine, Biomedical Research Institute Chonbuk National University Medical School and Hospital, Jeonju, Jeonbuk, Republic of Korea; ^2^ Department of Infectious Disease, Yanbian University Hospital, Yanji, Jilin Province, China

**Keywords:** OIP5, miR-15b-5p, hepatocellular carcinoma, tumor growth, metastasis

## Abstract

Opa interacting protein 5 (OIP5) is upregulated in some types of human cancers, but the biological implications of its upregulation have not yet been clarified in human hepatocellular carcinoma (HCC). In this study, the signaling pathway downstream of OIP5 was analyzed by proteome kinase profiling. A putative microRNA targeting OIP5 was identified using a miRNA PCR array. Tumorigenicity and metastatic ability were examined in an orthotopic animal model. OIP5 expression was strongly detected in early and advanced tumors via gene expression profiling and immunohistochemical staining analyses. Cells with knockdown of OIP5 via target shRNA exhibited reduced hepatic mass formation and metastatic tumor nodules in an orthotopic mouse model. OIP5-induced AKT activation was mediated by both mTORC2 and p38/PTEN activation. AKT activation was linked to mTORC1 and GSK-3β/β-catenin signaling, which are primarily associated with tumor cell growth and metastasis, respectively. miR-15b-5p, which targets OIP5, efficiently inhibited OIP5-mediated mTORC1 and GSK-3β/β-catenin signaling. These findings suggest that OIP5 may be involved in HCC growth and metastasis and that miR-15b-5p inhibits OIP5-mediated oncogenic signaling in HCC.

## INTRODUCTION

Opa interacting protein 5 (OIP5) encodes a 25-kDa protein with a coiled-coil domain that was found by yeast two-hybrid analysis to interact with Opa proteins [1]. *OIP5* is also called *Mis18beta* and *LINT-25*. *Mis18beta* is essential for the structure and function of the centromere/kinetochore, and accumulates specifically at telophase-G1 centromeres [2], forming a complex with C21orf45 and M18BP1. This protein also interacts with the retinoblastoma protein and regulates cell cycle progression via the E2F-Rb pathway [3]. OIP5 has been reported to be a testis-specific gene involved in gastric cancer [4]. In the fission yeast *Schizosaccharomyces pombe*, overexpression of OIP5 causes multi-septa formation and growth defects, both of which are considered cancer-related phenotypes. In addition, transient expression of OIP5 in NIH3T3 cells results in an increase in proliferation rate, highlighting its oncogenic properties [5]. Recently, OIP5 has also been reported to be upregulated in the tumors of colorectal cancer patients [6] and in female acute myeloid leukemia patients [7], implicating the protein as a potential therapeutic target for cancer [8]. Furthermore, it is a promising target for the development of new prognostic biomarkers and anti-cancer drugs in lung and esophageal cancers [9]. Despite the availability of a considerable amount of data, the precise function of OIP5 in human cancer, particularly hepatocellular carcinoma (HCC), remains unclear.

MicroRNAs (miRNAs) are endogenously expressed non-coding RNA oligonucleotides 21–23 bases in length that bind through topical sequence homology to the 3' untranslated region (UTR) of target messenger RNAs (mRNAs) and suppress gene expression [10]. Recent studies have implicated miR-15b regulation of proliferation and apoptosis in human osteoblastic cells and glioma cells through their targets, cyclin D1 and cyclin E [11, 12].

In the present study, overexpression of OIP5 was found to be associated with HCC malignancy through activation of the AKT/mTORC1 and β-catenin signaling pathways. In addition, miR-15b-5p efficiently inhibited OIP5-mediated oncogenic signaling.

## RESULTS

### OIP5 expression is mainly associated with tumor cell growth in HCC tissues and cell lines

**Figure 1:**
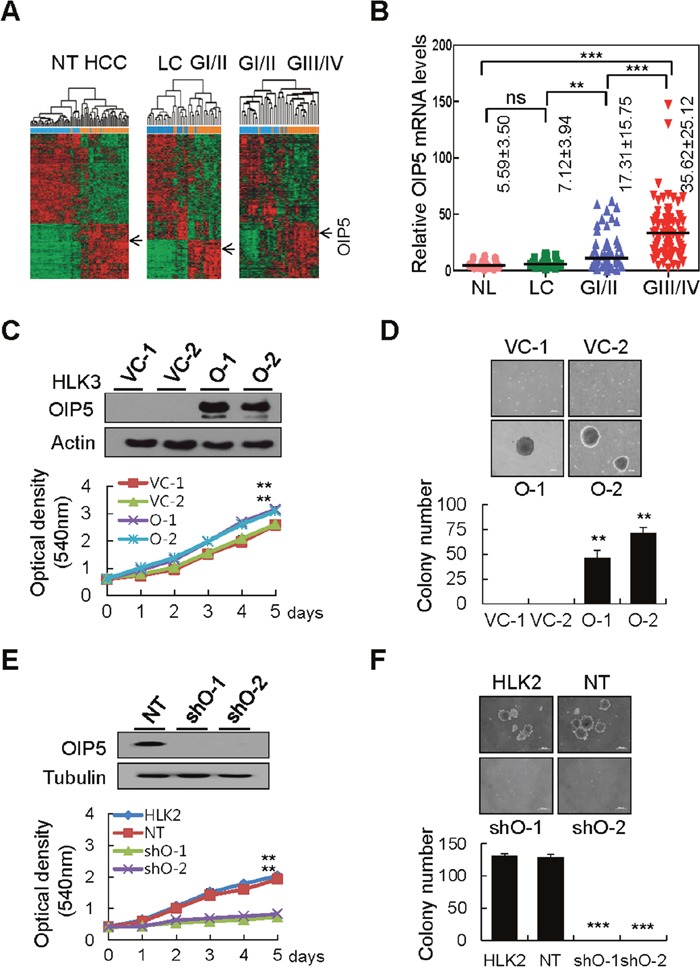
OIP5 expression in HCC tissues and cell lines modulates tumor cell growth **A**. Unsupervised hierarchical clustering separated the samples into two main groups: a non-tumor group (NT; normal liver + liver cirrhosis, n = 42) and an HCC group (GI/II + GIII/IV, n = 42). Two subgroups were also present: a liver cirrhosis group (LC, n = 21) and a well-differentiated HCC group (GI/II, n = 21); a well-differentiated HCC group (GI/II, n = 21) and a poorly differentiated HCC group (GIII/IV, n = 21). OIP5 was a unique gene with a two-fold or greater difference in expression from the mean at *P* < 0.05 based on the *t*-test for hierarchical clustering analysis. **B**. OIP5 mRNA levels were determined by real-time RT-PCR in four relevant groups of samples: normal liver (NL, n = 16), liver cirrhosis (LC, n = 19), Edmondson grade I/II (GI/II, n = 58), and Edmondson grade III/IV (GIII/IV, n = 74). Bars indicate medians. Values represent mean ± SD. *P* values represent the results of Mann-Whitney U tests. The Kruskal-Wallis test was used for overall comparisons. **P < 0.01; ***P < 0.001. **C**. OIP5 expression in HLK3 cells (O) stably transfected with OIP5 expression plasmid evaluated via Western blot (upper panels). The proliferation of OIP5-expressing transfectants was evaluated by MTT assay (lower panels). Absorbance of the solution was measured at 540 nm. Triplicate experiments with quadruplicate samples were performed. The values represent the mean ± SD. **P < 0.01. VC, vector control. **D**. Soft agar colony formation assay on OIP5-expressing HLK3 cells. The colonies shown are two weeks old. Scale bar: 200 μm (upper panels). Quantification of colony formation (lower panels). Each bar represents the mean ± SD (n = 3). **P < 0.01. **E**. Knockdown of OIP5 (shO) by lentiviral delivery of OIP5 shRNA, evaluated by Western blot (upper panels). The proliferation of HLK2 cells with OIP5 knockdown was evaluated by MTT assay (lower panels). **P < 0.01. NT, nontarget. **F**. Soft agar colony formation assay of HLK2 cells with OIP5 knockdown (upper panels). Scale bar: 200 μm. Quantification of colony formation (lower panels). Each bar represents the mean ± SD (n = 3). ***P < 0.001.

Microarray data derived from the Gene Expression Omnibus (GEO) database (GSE36411), containing 42 HCCs and corresponding non-tumor tissues, revealed that OIP5 expression in HCC tissue was significantly higher than expression in matched non-tumor tissues surrounding the liver (Figure 1A). In the two major sample clusters, genes from a non-tumor liver tissue group (NT; normal liver + liver cirrhosis) and a tumor tissue group (HCC; Edmondson grades I to IV) with a *P* <0.05 and a mean difference of expression > 1.5 between the two groups were selected by unsupervised hierarchical clustering analysis. Next, using the same clustering analysis of the three subgroups (liver cirrhosis [LC], well-differentiated HCC [Edmondson grade I/II], and poorly-differentiated HCC [Edmondson grade III/IV]), we found that *OIP5* expression was significantly higher in GI/II HCC than in LC, and was higher in GIII/IV HCC than in GI/II HCC, implicating upregulation of *OIP5* in HCC progression. We further statistically analyzed *OIP5* mRNA levels via real-time RT-PCR in four groups of samples from the independent HCC cohorts, NL, LC, GI/II, and GIII/IV (Figure 1B). The level of *OIP5* mRNA significantly increased with worsening differentiation status, lack of fibrous capsule formation, microvessel invasion, intrahepatic metastasis, and advanced HCC stage (Supplementary Table 1).

A polyclonal rabbit antibody to OIP5 was tested for specific immunoreactivity by transfecting HEK293T cells with GFP- or c-Myc-tagged expression plasmids (Supplementary Figure 1A). OIP5 was highly expressed in HCC (75%) compared with non-tumor tissue, in 12 HCC/non-tumor tissue pairs (Supplementary Figure 1B). Immunohistochemical (IHC) staining for OIP5 in various HCC tissues revealed that OIP5 was moderately expressed in tumors compared to the much lower expression levels observed in surrounding non-tumor and normal liver tissues (Supplementary Figure 1C). OIP5 immunoreactivity was localized mainly in the nucleus, and less so in the cytoplasm of HCC cells. OIP5 was highly expressed in HepG2, Huh7, HLK2, and HKK2 cells, but was weakly or barely expressed in immortalized hepatocytes and other HCC cells (Supplementary Figure 1D). Immunofluorescence assays revealed that GFP-tagged OIP5 overlapped with OIP5 immunoreactivity and was prominently localized in the nucleus, and less abundant in the cytoplasm of HLK3 and HepG2 cells (Supplementary Figure 2A). An MTT assay revealed that the growth rate of HLK3 cells stably expressing OIP5 was greater than that of vector-control cells (Figure 1C). Accordingly, a colony generation assay revealed that OIP5 overexpression increased colony number by more than two-fold that of vector-control cells (Supplementary Figure 2B). In contrast, when OIP5 was stably knocked down in HLK2 cells using two different small hairpin RNA (shRNA) constructs, OIP5 silencing remarkably reduced colony numbers compared with control cells (Supplementary Figure 2C). We also examined the effect of OIP5 overexpression on the colony-forming capabilities of HLK3 and SH-J1 cells, and found that OIP5 expression significantly increased colony formation in soft agar compared with that by control cells (Figure 1D and Supplementary Figure 2D). MTT assay showed that the proliferation rate of HLK2 cells with OIP5 knockdown was prominently reduced (Figure 1E). Furthermore, anchorage-independent cell growth was barely detectable in HLK2 and Huh7 cells with OIP5 knockdown (Figure 1F and Supplementary Figure 2E). These results collectively demonstrated that OIP5 promotes the proliferation of HCC cells.

### OIP5-induced tumor growth and metastasis *in vitro* and *in vivo*

**Figure 2:**
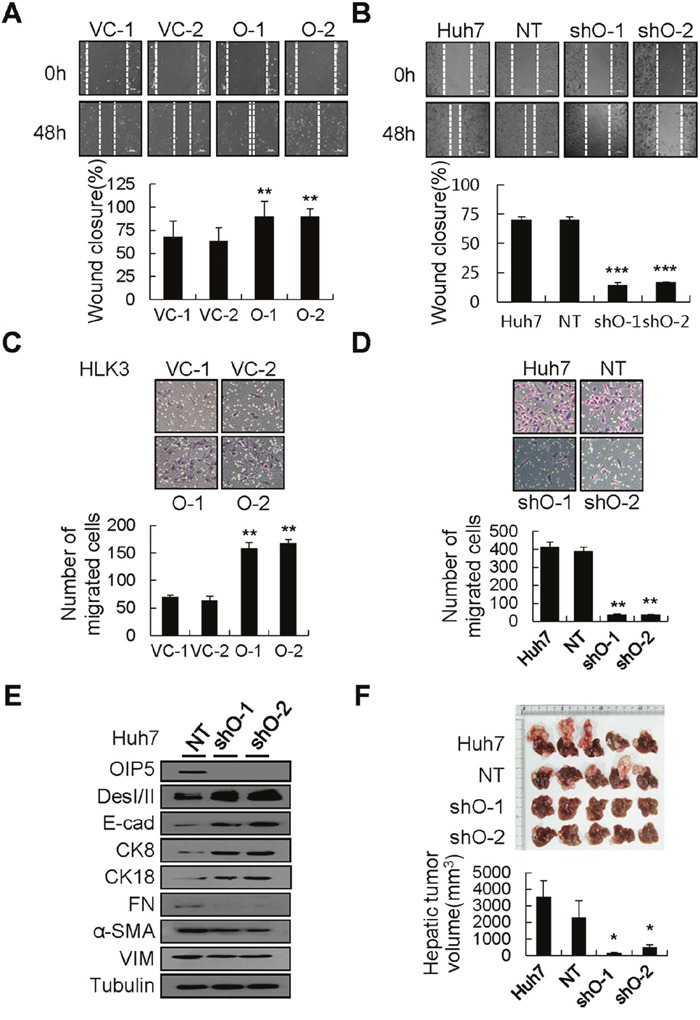
Modulation of migration and invasiveness by OIP5 **A**. Phase contrast images of HLK3 cells stably expressing OIP5 were taken 48 h after transduction in order to assess cell migration (upper panels). The results shown are representative of three independent experiments. Quantitative measurements of wound closure ability are shown (lower panels) (n = 3, mean ± SD). **P < 0.01. **B**. Migratory ability was determined in Huh7 cells transduced with lentivirus encoding OIP5 shRNA via a wound healing assay. Phase contrast images were taken 48 h after transduction in order to assess cell migration (upper panels). The results shown are representative of three independent experiments. Quantitative measurements of wound closure ability are shown (lower panels) (n = 3, mean ± SD). ***P < 0.001. **C**. Numerous stable OIP5 transfectants, but only a few vector control cells, had traversed the Matrigel-coated membrane after 48 h (upper panels). The cells that invaded were normalized to the viable cell mass on both sides of the membrane. Quantitative measurements of invasiveness are shown (lower panels) (n = 3, mean ± SD). **P < 0.01. **D**. Photomicrographs of a modified Boyden chamber assay of Huh7 cells with OIP5 knockdown. The cells traversed the Matrigel-coated membrane after 48 h (upper panels). Cells that invaded were normalized to the viable cell mass on both sides of the membrane. Quantitative measurements of invasive ability are shown (lower panels) (n = 3, mean ± SD). **P < 0.01. **E**. Western blot analyses showed loss of mesenchymal markers and acquisition of epithelial marker proteins in Huh7 cells with OIP5 knockdown compared with non-target (NT) cells. The results shown are representative of three independent experiments. DesI/II, desmoplakin I/II; E-cad, E-cadherin; CK, cytokeratin; FN, fibronectin; α-SMA, alpha smooth muscle actin; VIM, vimentin. **F**. Mass-forming tumors and nodules in the liver of mice three weeks after intrahepatic inoculation of cells with or without OIP5 knockdown (upper panels). Quantification of nodules in the livers of mice (lower panels). Each bar represents the mean ± SD. *P < 0.05.

To investigate the molecular mechanisms of OIP5-mediated metastasis in HCC, cell migration ability was analyzed via wound healing assay. HLK3 cells stably expressing OIP5 showed faster wound closure than did vector control cells (Figure 2A). In addition, OIP5 knockdown cells were significantly less likely to migrate to a wounded area than were parent or non-target control Huh7 and HLK2 cells (Figure 2B and Supplementary Figure 3A). In a Matrigel invasion chamber assay, OIP5 expression penetrated the matrix and colonized the bottom surface of the Matrigel-coated membrane to a larger extent than did vector control cells (Figure 2C). In contrast, Huh7 and HLK2 cells with OIP5 knockdown reduced cell invasiveness (Figure 2D and Supplementary Figure 3B). Collectively, OIP5 expression enhanced cell migration and invasiveness, whereas OIP5 suppression appeared to inhibit the migration and invasiveness of tumor cells *in vitro*. Finally, we examined changes in EMT marker expression in Huh7 cells with OIP5 knockdown. OIP5 knockdown substantially decreased the expression of mesenchymal markers (FN, α-SMA, and VIM), and increased the expression of epithelial markers (DesI/II, E-cad, CK8, and CK18) (Figure 2E). OIP5 expression affected the expression of EMT markers and vice versa (Supplementary Figure 3C). These results suggest that OIP5 acts upstream of EMT changes.

Huh7 cells with OIP5 knockdown were orthotopically inoculated into the left lobe of mouse livers to examine whether OIP5 affects tumorigenicity. Mass-forming tumors with intrahepatic tumor nodules were easily detected in the livers of mice injected with parent or non-target control cells, whereas only a few intrahepatic tumor nodules were found in mice injected with OIP5-knockdown cells (Figure 2F). OIP5 immunoreactivity was barely detected in hepatic tumors derived from cells with OIP5 knockdown (Supplementary Figure 4A). In addition, numerous mesenteric nodules were found in mice injected with parent or non-target control cells, whereas fewer mesenteric nodules were found in mice injected with OIP5-knockdown cells (Supplementary Figure 4B). These results suggest that OIP5 is substantially involved in tumor growth as well as in metastasis.

### OIP5 expression during the mitotic phase in HCC cells

**Figure 3:**
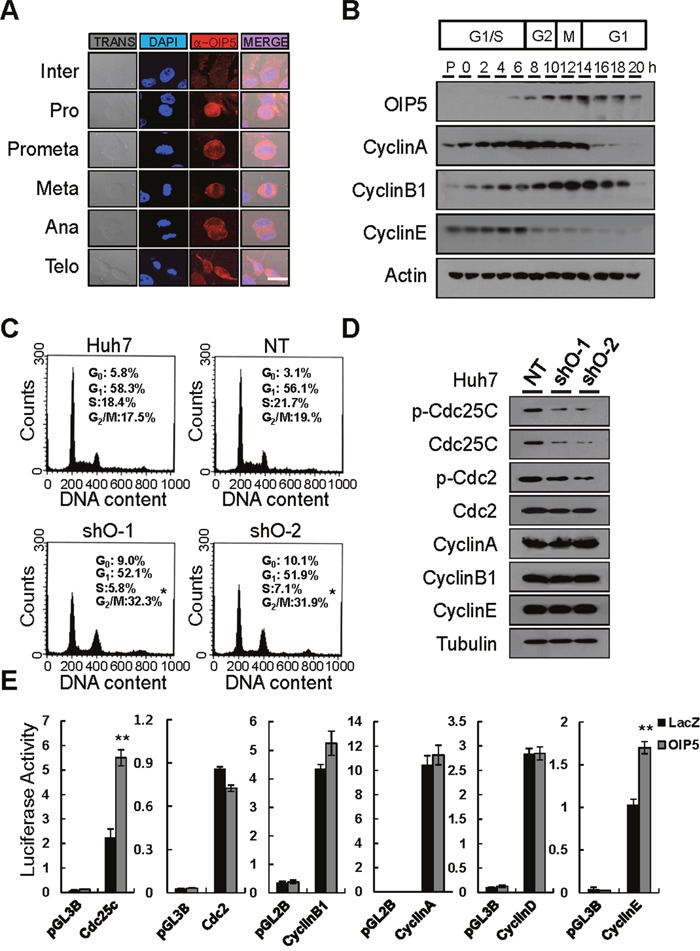
Analysis of the expression of OIP5 during cell cycle progression **A**. Indirect immunofluorescence microscopy of HLK3 cells during cell cycle progression. Endogenous OIP5 expression at various stages of mitosis. Inter, interphase; Pro, prophase; Met, metaphase; Ana, anaphase; Telo, telophase. Trans, transmission. Bar, 20 μm. **B**. HeLa cells were synchronized in G_1_ using a thymidine/aphidicolin double block and then released. Samples were collected for immunoblot analysis at 2 h intervals after release. The cells were extracted and probed with cyclin A, cyclin B1, cyclin E, and OIP5 antibodies. **C**. Cell cycle distribution was analyzed using flow cytometry after staining with propidium iodide (PI). Huh7 cells were transduced with lentiviruses carrying shRNA-targeting OIP5 (shO-1 and shO-2) as well as non-targeting shRNA (NT). *P < 0.05. (n = 3). **D**. Representative immunoblots showing that OIP5 knockdown changed the expression of Cdc2 and Cdc25C, but not the expression of cyclin A, cyclin B1, or cyclin E in Huh7 cells. The immunoblot was probed with the indicated antibodies. Tubulin was used as a loading control. **E**. Promoter activities of cyclins and cyclin-associated proteins in response to OIP5 expression. Two independent quadruplicate experiments were performed. The values represent the mean ± SD. **P < 0.01.

OIP5 immunoreactivity was prominent in mitotic cells. Therefore, we analyzed OIP5 expression in mitosis. OIP5 overexpression began in pre-prophase. After anaphase and telophase, its expression was reduced to its nadir during interphase (Figure 3A). A thymidine-aphidicolin double-block experiment revealed cell cycle-dependent expression of OIP5 during the G_2_/M-phase and early G_1_ phase, which followed cyclin E expression (Figure 3B). Next, to determine the exact role of OIP5 in cell cycle progression, cell cycle analysis revealed that OIP5 knockdown in Huh7 cells resulted in G_2_/M arrest (Figure 3C). To further examine this cell cycle arrest, levels of G_2_/M cell cycle-related proteins, such as Cdc2, Cdc25C, cyclin A, cyclin B1, and cyclin E, were examined by Western blotting (Figure 3D). The results indicated that phosphorylation and expression of Cdc2 and Cdc25C were suppressed by the knockdown of OIP5. The Cdc2/cyclin B1 complex is essential for the G_2_/M transition, and cyclin B1 is also important for Cdc2 activity [13]. However, knockdown of OIP5 did not change the levels of cyclin A, cyclin B1, or cyclin E. Next, we determined whether OIP5 modulates activity of the promoters of cyclins and their associated proteins (Figure 3E). OIP5 prominently upregulated the activity of the Cdc25C promoter, but did not change the activity of the Cdc2 promoter, suggesting that OIP5 regulates these proteins transcriptionally or post-transcriptionally. In addition, to determine the molecular mechanism of OIP5 in cell death, we analyzed apoptotic cell death via flow cytometry in Huh7 cells with OIP5 knockdown (Supplementary Figure 5). OIP5 suppression significantly induced more apoptotic cell death than the nontarget control.

### Downstream AKT activation by OIP5

**Figure 4:**
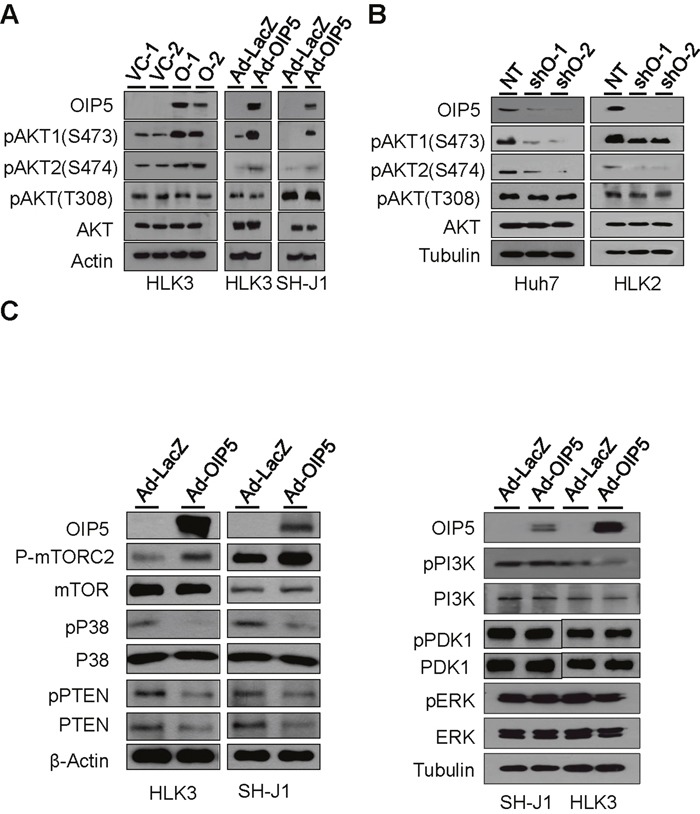
Phosphorylation of AKT by OIP5 **A**. Phosphorylation of AKT in stable OIP5 transfectants of HLK3 cells compared to vector control cells, and in HLK3 and SH-J1 cells transiently infected with Ad-OIP5 compared to Ad-LacZ control cells. **B**. Change in AKT phosphorylation in Huh7 and HLK2 cells by OIP5 knockdown via lentiviral delivery of shRNA. **C**. Activation of mTORC2, and inactivation of p38 and PTEN in HLK3 or SH-J1 cells transiently infected with Ad-OIP5 or Ad-LacZ adenovirus (n = 3). Activation levels of PI3K, PDK1, and ERK did not change in SH-J1 or HLK3 cells transiently infected with Ad-OIP5 or Ad-LacZ adenovirus (n = 3).

To explore the mechanisms of OIP5 oncogenicity, we examined the activation of its downstream molecules using human phospho-MAPK arrays on lysates of HLK3 cells infected with adenovirus Ad-GFP/OIP5 or Ad-LacZ (Supplementary Figure 6A and 6B). Intriguingly, phosphorylation of AKT1 (S473), AKT2 (S474), and AKT3 (S472) was observed, as was panAKT phosphorylation. Transient expression of OIP5 by infection with Ad-OIP5 also increased the phosphorylation of AKT1 and AKT2 in HLK3 and SH-J1 cells (Figure 4A). Next, we observed that OIP5 knockdown resulted in a decrease in AKT1 and AKT2 phosphorylation in Huh7 and HLK2 cells (Figure 4B). Furthermore, immunofluorescence assay revealed that the cellular localization of OIP5 seemed to occur predominantly in the nucleus, whereas that of AKT occurred mainly in the cytoplasm of cells (Supplementary Figure 6C). Next, we determined whether OIP5 is involved in the activation of mTORC2, p38, PTEN, PI3K, PDK1, or ERK1/2 (Figure 4C), as activation of the PI3K/AKT and MAPK pathways is commonly implicated in HCC [14, 15]. We found that OIP5 increased mTORC2 activation and decreased p38 and PTEN activation compared to mock expression. However, this did not alter activation of PI3K, PDK1, and ERK molecules. Inactivation of p38 results in decreased expression of PTEN, which is linked to AKT activation [16]. Therefore, p38 MAPK/PTEN and mTORC2 may be independently involved in AKT activation.

### Nuclear translocation of β-catenin by AKT activation

**Figure 5:**
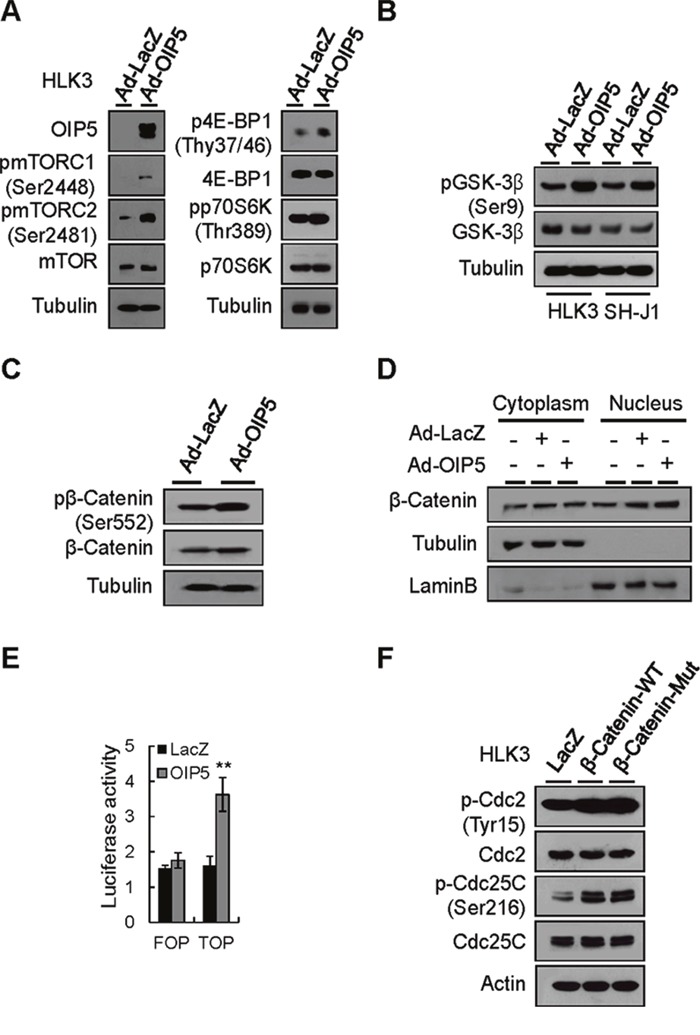
Activation of the mTORC1 and β-catenin signaling pathways by OIP5 **A**. Activation of mTORC1 in HLK3 cells transiently infected with Ad-OIP5 compared to Ad-LacZ control cells on Western blot analysis. **B**. Phosphorylation of GSK-3β by OIP5 in HLK3 and SH-J1 cells transiently infected with Ad-OIP5 or Ad-LacZ adenovirus (n = 3). **C**. Phosphorylation of β-catenin at the S552 site by OIP5 in HLK3 cells transiently infected with Ad-OIP5 or Ad-LacZ adenovirus (n = 3). **D**. Immunoblot analysis of nuclear and cytoplasmic levels of β-catenin in HLK3 cells transiently infected with Ad-OIP5 or Ad-LacZ adenovirus (n = 3). **E**. TCF/LEF-dependent transcriptional activity of β-catenin in HEK293T cells transfected with TOP/FOP flash reporter plasmids. Assays of relative luciferase activity in cells were performed (n = 3). Each bar represents mean ± SD. **P < 0.01. **F**. Changes in Cdc2 and Cdc25C activation by β-catenin in HLK3 cells. Immunoblots were probed with the indicated antibodies.

AKT activation occurs upstream of mTORC1 activation or downstream of mTORC2 [17, 18]. Accordingly, OIP5 expression efficiently results in the phosphorylation of mTORC2, mTORC1, 4E-BP1, and p70S6K in HLK3 cells (Figure 5A). AKT can regulate β-catenin-dependent transcription either by inhibiting GSK-3β or by directly phosphorylating and activating β-catenin [19, 20]. To further determine the contribution of AKT activation to increased β-catenin signaling, we examined the phosphorylation levels of GSK-3β and β-catenin in HLK3 and SH-J1 cells after transient OIP5 expression by transduction with Ad-OIP5 (Figure 5B and 5C). OIP5 expression significantly increased the phosphorylation levels of GSK-3β and β-catenin. Immunoblot analysis revealed that increased β-catenin expression was confined to the nuclear fraction of cells transduced with Ad-OIP5, and was less apparent in the cytoplasmic fraction of these cells (Figure 5D). Ectopic OIP5 expression enhanced the nuclear translocation of endogenous β-catenin (Supplementary Figure 7). Accordingly, OIP5 significantly increased TOP luciferase reporter activity (Figure 5E). Activated and mutant β-catenin prominently enhanced the phosphorylation of Cdc2 and Cdc25C and intriguingly increased the transcript level of Cdc25C (Figure 5F).

### OIP5-mediated tumor growth and metastasis via mTORC1 and β-catenin

**Figure 6:**
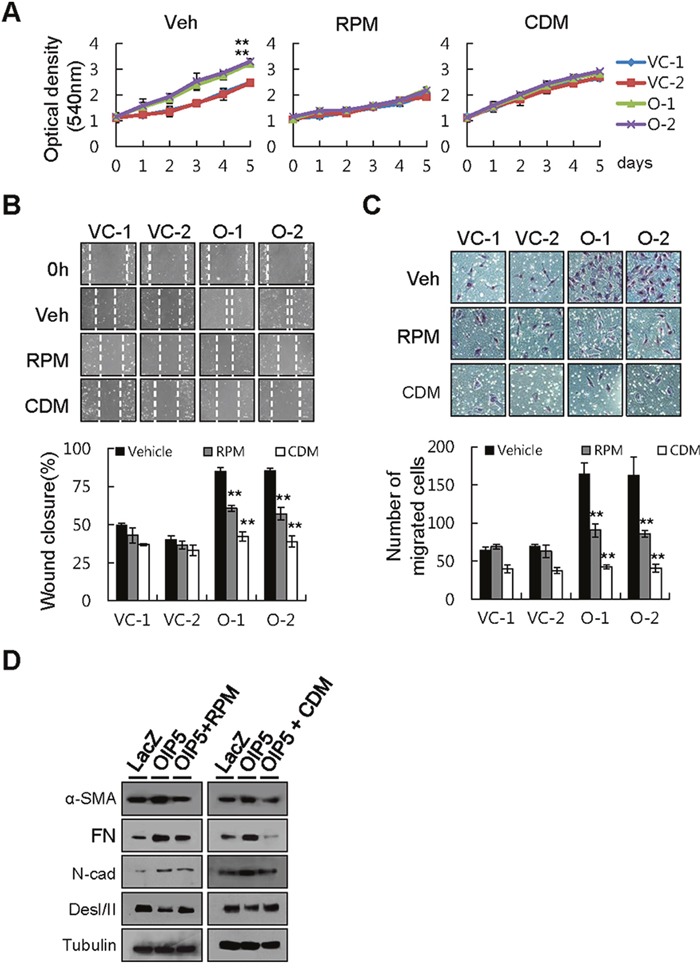
Inhibition of tumor cell growth and metastatic ability by rapamycin or cardamonin **A**. Growth inhibition of OIP5 transfectants treated with rapamycin (RPM, 100 nM), cardamonin (CDM, 10 μM), or vehicle (Veh). Cell growth was measured by MTT assay five days after the cells were seeded on 24-well plates (2 × 10^4^ cells/well). Each value represents the mean ± SD. **P < 0.01. **B**. A wound healing assay demonstrated substantial inhibition of OIP5-mediated wound closure by RPM (100 nM) or CDM (10 μM) (upper panels). Quantitative measurements are shown (lower panels). Each bar represents the mean ± SD (n = 3). **P < 0.01. **C**. A Matrigel assay showed that the invasiveness of OIP5 transfectants was inhibited by CDM (10 μM), but less so by RPM (100 nM) or vehicle (upper panels). Quantitative measurements are shown (lower panels). Each bar represents the mean ± SD (n = 3). **P < 0.01. **D**. Change in EMT marker protein expression by treatment with either RPM or CDM in HLK3 cells transiently infected with Ad-OIP5 or Ad-LacZ adenovirus (n = 3).

To examine the role of these pathways in tumor cell growth and metastatic ability, we measured cell growth in OIP5-expressing HLK3 cells treated with the mTORC1 inhibitor rapamycin (RPM, 100 nM) or the β-catenin inhibitor cardamonin (CDM, 10 μM). RPM substantially inhibited OIP5-dependent tumor cell growth, but had a smaller effect on OIP5-independent tumor cell growth (Figure 6A). CDM only partially inhibited OIP5-dependent tumor cell growth. RPM treatment induced significant G_1_/S arrest, while CDM treatment caused G_2_/M arrest (Supplementary Figure 8A). We also analyzed the expression or activation of cyclins and their associated proteins in HLK3 cells after treatment with either RPM or CDM following transient OIP5 expression by transduction with Ad-OIP5. RPM increased p21 in Ad-OIP5-infected HLK3 cells, despite decreasing CDK2, CDK4, and CDK6 expression in the same cells (Supplementary Figure 8B). CDM significantly decreased the expression and phosphorylation of Cdc25C, and increased cyclin B1 expression, in Ad-OIP5-infected HLK3 cells (Supplementary Figure 8C). In a wound healing assay, CDM largely inhibited both OIP5-mediated wound closure (Figure 6B) and OIP5-mediated transwell invasion ability (Figure 6C). RPM, however, caused only partial inhibition of wound repair and invasion ability. These results suggest that the AKT/mTORC1 pathway plays a major role in regulating tumor cell growth, while the AKT/β-catenin pathway is involved in modulating metastatic ability. However, the AKT/mTORC1 and AKT/β-catenin pathways also appeared to have partially overlapping functions. Finally, we evaluated changes in the level of the OIP5-mediated EMT marker via treatment with either RPM or CDM (Figure 6D). RPM and CDM similarly inhibited the expression of OIP5-mediated mesenchymal markers, including α-SMA, FN, and N-cadherin, while increasing the expression of the epithelial marker Des I/II.

### OIP5 is a direct target of miR-15b-5p

**Figure 7:**
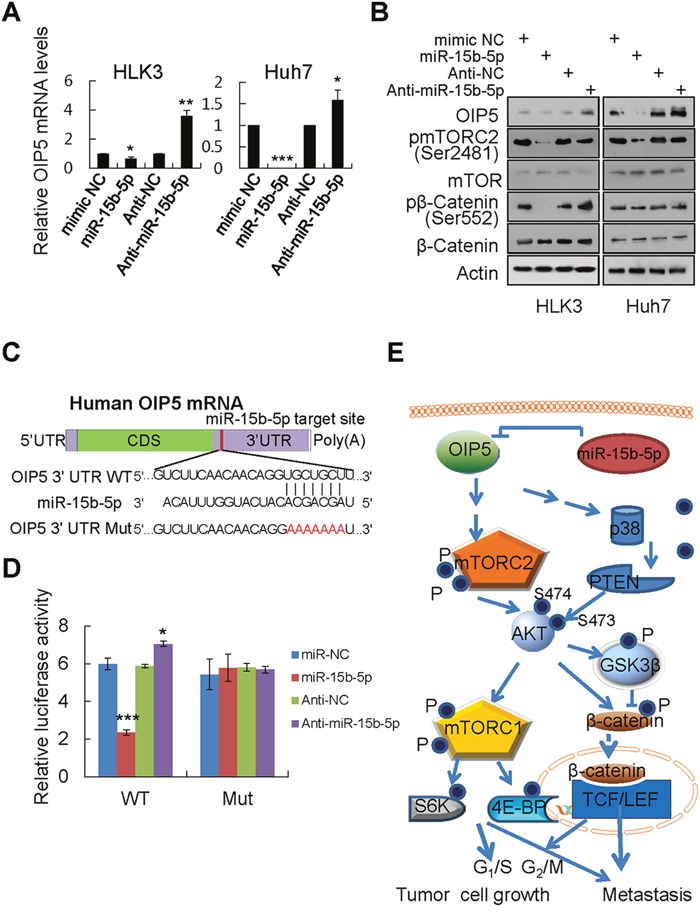
OIP5 is a direct target of miR-15b-5p **A**. HLK3 and Huh7 cells transiently transfected with 5 nM miR negative control (NC) or miR-15b-5p mimics, and 50 nM anti-miR negative control (Anti-NC) or miR-15b-5p inhibitor, for 48 h. Decreased OIP5 mRNA levels were observed in HLK3 and Huh7 cells transfected with the miR-15b-5p mimic, while increased OIP5 mRNA expression was observed in HLK3 and Huh7 cells transfected with miR-15b-5p inhibitor. *P < 0.05; **P < 0.01; ***P < 0.001. **B**. Effects of miR-15b-5p on OIP5 expression and on the activation of downstream molecules. **C**. Wild type and mutant putative miR-15b-5p binding sequence in the 3'UTR of OIP5 mRNA. **D**. Relative luciferase activity was significantly decreased by cotransfection of wild type (WT) pGL3-OIP5 3'UTR with miR-15b-5p mimics or anti-miR-15b-5p into HEK293T cells, but not by cotransfection of a mutant (Mut) pGL3-OIP5 3'UTR with miR-15b-5p mimics. ***P < 0.001. **E**. Schematic model of the upstream (mTORC2 and PTEN) and downstream (mTORC1 and β-catenin) signaling pathways of AKT in OIP5 expressed cells.

To identify miRNAs whose expression correlates negatively with OIP5 expression, we performed an miRNA PCR array and identified miRNAs that were differentially expressed between low OIP5-expressing HLK3 cells and high OIP5-expressing Huh7 cells (Supplementary Figure 9A). Twenty-five miRNAs had lower expression in the HCC Huh7 cell line than in the immortalized HLK3 cell line (Supplementary Table 2). Of these, 15 miRNAs had significantly lower expression (> 2-fold, *P* < 0.05; Supplementary Figure 9B). Using TargetScan and the miRanda database, we identified miR-15b-5p as a potential OIP5 regulator in HCC cells (Supplementary Figure 9C). Only miR-15b-5p was relatively elevated in HLK3 cells and lower in Huh7 cells. Furthermore, whether endogenous OIP5 is regulated by miR-15b-5p in other HCC cells was investigated. Real-time PCR revealed that OIP5 mRNA levels were inversely correlated with miR-15b-5p levels in 3 immortalized liver cell lines and 13 HCC cell lines (Supplementary Figure 9D). Accordingly, mRNA and protein levels of OIP5 were measured in HLK3 and Huh7 cells following transfection with miR-15b-5p mimic and anti-miR-15b-5p. OIP5 mRNA and protein levels were decreased in HLK3 and Huh7 cells transfected with miR-15b-5p overexpression, while increased OIP5 mRNA and protein levels were observed in cells transfected with miR-15b-5p silencing (Figure 7A and 7B). miR-15b-5p also regulated OIP5 downstream protein mTORC1 and β-catenin activation. Using the miRNA database, we found that miR-15b-5p is predicted to bind to the OIP5 3'UTR (Figure 7C). A dual-luciferase assay demonstrated a significant reduction in luciferase activities after cotransfection of miR-15b-5p and the wild type pGL3-OIP5 3'UTR reporter, while a significant increase in luciferase activity after cotransfection of anti-miR-15b-5p and the wild-type pGL3-OIP5 3'UTR reporter, but not after cotransfection of a mutant pGL3-OIP5 3'UTR reporter (Figure 7D). Next, we measured miR-15b-5p levels in cells during the cell division cycle and found they were inversely regulated in a cyclic manner compared to OIP5 mRNA levels (Supplementary Figure 10). These results demonstrated that miR-15b-5p directly regulates OIP5. The schematic model demonstrated that OIP5 regulates tumor growth and metastasis through AKT activation, which is mediated by both mTORC2 activation and p38/PTEN inactivation. AKT activation is linked to the downstream signaling pathway of both mTORC1 and β-catenin activation (Figure 7E).

## DISCUSSION

OIP5 is highly expressed during the mitotic phase of the cell cycle. OIP5 expression is increased in pre-prophase and peaks in metaphase, while OIP5 knockdown mediates cell cycle arrest in the G_2_/M phase and apoptotic cell death. This knockdown experiment demonstrated that OIP5 was associated with the regulation and activation of cyclin-dependent kinase Cdc2 and phosphatase Cdc25C. ERK-MAP kinases were recently reported to be directly involved in activating Cdc25C during the G_2_/M transition [21]. The simultaneous activation of the MAP kinase and AKT pathways in HBV-replicating hepatocytes resulted in dysregulation of cell cycle control, leading to malignant transformation associated with chronic hepatitis B infection [22]. In the present study, OIP5 expression enhanced AKT activation through the mTORC2 and p38/PTEN signaling pathways. These results suggest that OIP5 expression is correlated with the mitotic proliferation of tumor cells, and that OIP5 knockdown-mediated inhibition of tumor cell growth causes accumulation of G_2_/M phase cell cycle regulators via OIP5/AKT signaling. Interestingly, our data also demonstrated that OIP5 knockdown induced apoptotic cell death, as reported previously [23].

Activation of AKT results in the phosphorylation of a number of downstream substrates, such as glycogen synthase kinase (GSK3), Bad, caspase 9, and forkhead transcription factors Raf, IκB kinase, and phosphodiesterase 3B [24]. GSK3, one of the principal physiological substrates of AKT, is a ubiquitously expressed serine/threonine protein kinase that was initially identified as an enzyme that regulates glycogen synthesis in response to insulin [25, 26]. GSK-3β plays an important role in the WNT pathway by regulating the degradation of β-catenin [27, 28]. WNT signaling is implicated in proliferation, differentiation, embryogenesis, and tumorigenesis [29]. β-catenin is then translocated into the nucleus where it acts as a transcription factor for several genes, including Myc and cyclin D1 [30]. In HCC, one of the major causes of WNT activation is β-catenin mutation with a rate between 20 and 40% [31, 32]. However, our data show that OIP5 enhances the nuclear translocation of β-catenin through the phosphorylation of GSK-3β at S9 and of β-catenin at S522. Consequently, OIP5 activates β-catenin signaling.

AKT induction also triggers activation of the mTORC1/p70S6K cascade, a pathway that contributes to cell growth and survival [33]. mTOR is activated both via phosphorylation by AKT and through inactivation of mTOR inhibitor TSC2. Although the activation of mTOR is limited to a subset of human HCCs, AKT deregulation has a pivotal role in human liver cancer [34]. Accordingly, the activation of the AKT/mTORC1 pathway in the livers of mice induces lipogenesis and tumor development [35]. We initially proposed that OIP5 expression itself can activate the AKT/mTORC1 pathway, which prominently contributes to tumor cell proliferation in association with G_1_/S cell cycle progression and, to a lesser extent, tumor metastasis. OIP5 expression resulted in phosphorylation of AKT at S473 and S474, which is linked to activation of the mTORC1 pathway. In addition, there is evidence of anti-tumor activity following mTOR blockade after treatment with rapamycin and its analogs in experimental models of HCC [36, 37].

miR-15 acts on proteins regulating cell cycle regulation, proliferation, and stem cell renewal [38]. miR-15b is a cell cycle regulator in glioma [12]. Our present study showed that miR-15b-5p directly binds to the 3'UTR of OIP5 and downregulates its mRNA and protein expression. miR-15b-5p was inversely regulated in a cyclic manner compared with OIP5 mRNA levels, and plays a role in the inhibition of oncogenic signaling of OIP5 through targeting mTORC1 and β-catenin, as well as functioning as a tumor suppressor.

In summary, OIP5 seems to be involved in HCC growth and metastasis through the mTORC1 and β-catenin signaling pathways. miR-15b-5p efficiently inhibits OIP5-mediated oncogenic signaling in HCC. Therefore, OIP5 may serve as a novel therapeutic target in HCC.

## MATERIALS AND METHODS

### Tissue acquisition

Surgically-resected HCC and matched non-tumor tissues were collected according to a protocol conforming to the ethical guidelines of the Institutional Review Board (IRB) of Chonbuk National University Hospital. The Research Ethics Committee of Chonbuk National University Hospital approved the study. Written informed consent was obtained from each patient. Pathologists histologically confirmed HCC and non-tumor tissues.

### Cell lines

HCC cell lines, including HepG2, Hep3B, SK-HEP-1, and Alexander cells as well as immortalized cell lines such as Chang liver and THLE2 were procured from the ATCC (Manassas, VA, USA). Huh7 cells were acquired from the Korean Cell Line Bank (KCLB, Seoul, South Korea). HLK2, HKK2, HLK4, HLK1, HLK5, HKK1, HLK6, and SH-J1 cells as well as immortalized cell line HLK3 were established in our laboratory from surgical specimens of HCC patients [39]. The cells lines, with the exception of THLE2, were cultured at 37°C in a humidified atmosphere containing 5% CO_2_ in DMEM medium supplemented with 10% fetal bovine serum (FBS; Gibco-BRL, Carlsbad, CA, USA), 1% penicillin and streptomycin solution (Sigma), 3 mM taurine, and 25 mM HEPES (Invitrogen). THLE2 cells were cultured using a BEGM bullet kit (Clonetics, Walkersville, MD, USA).

### Real-time PCR

RNA prepared from dissected tissues was precipitated with isopropanol and dissolved in DEPC-treated distilled water. Reverse transcription (RT) was performed using 2 μg total RNA, 50 μM decamer, and 1 μl (200 units) RT-PCR Superscript II (Invitrogen) at 37°C for 50 min. Specific primers for *OIP5* were designed employing the Primerdepot website (http://primerdepot.nci.nih.gov/): *OIP5* (accession No. NM_007280), 5'- TGGCATTGAAGGTTCACTCA −3' (forward), and 5'- AGGGCAGCATGGGTAGAATA −3' (reverse). A control *18S* ribosomal RNA primer from Applied Biosystems was used as the invariant control. The real-time RT-PCR reaction mixture, which consisted of 10 ng reverse-transcribed total RNA, 167 nM forward and reverse primers, and 2 × PCR master mixture in a final volume of 10 μl, was placed in 384-well plates and analyzed using the ABI Prism 7900HT Sequence Detection System (Applied Biosystems, Foster City, CA, USA).

### Immunoblotting

To prepare whole cell lysates, cells were lysed on ice in RIPA lysis buffer (50 mM Tris-HCL [pH 7.4], 150 mM NaCl, 0.25% sodium deoxycholate, 1% NP-40, 1 mM EDTA, 0.1% sodium dodecyl sulfate [SDS], 1 mM PMSF, 10 μg/ml leupeptin, 10 μg/ml aprotinin). Thirty micrograms of protein of cell or tissue lysates was resolved by SDS-PAGE then transferred to a nitrocellulose membrane (Millipore, Billerica, MA, USA), which was then blocked with Tris-buffered saline solution containing 0.05% Tween 20 for 1 h at room temperature. The blots were then probed with the relevant antibodies overnight at 4°C, washed, and probed again with species-specific secondary antibodies coupled to horseradish peroxidase (Sigma). Immunoreactivity was assessed using an ECL prime Western blotting kit (Amersham Pharmacia Biotech, Buckinghamshire, UK), according to the manufacturer's instructions. For nuclear and cytosolic cellular fractionation, both fractions were isolated from HLK3 cells using the commercially available NE-PER Nuclear and Cytoplasmic Extraction Reagents from Thermo Scientific (Rockford, IL, USA), following the manufacturer's protocol. For the thymidine-aphidicolin double-block, 25% confluent HeLa cells were cultured in the presence of 2 mM thymidine for 14 h and released into fresh medium for 14 h. They were then incubated in the presence of 2 μg/ml aphidicolin for 14 h before release into fresh medium, as described previously [40].

Anti-human phospho-Akt (Ser473), AKT, phospho-GSK-3β (Ser9), phospho-Cdc25C (Ser216), phospho-Cdc2 (Tyr15), phospho-ERK, ERK, phospho-mTOR (Ser2448), phospho-mTOR (Ser2481), mTOR, phospho-4E-BP1(Thr37/46), 4E-BP1, phospho-p70s6k (Thr389), p70s6k, phospho-β-catenin (Ser552), phospho-PDK1 (Ser241), PDK1, phospho-PI3K (Y458/Y199), and PI3K antibodies were purchased from Cell Signaling Technology (Danvers, MA, USA). Polyclonal anti-human Cdc25C (C-20), Cdc2, cyclin A (H-432), cyclin B1 (H-433), cyclin D1 (C-20), cyclin E (C-19), GSK-3β (L-17), β-catenin (H-102), Lamin B (C-20), Cytokeratin 18 (C-04), Cytokeratin 8 (M20), N-cadherin (H69), Desmoplakin I/II (G20), c-Myc (9E10), GFP (FL), P21 (C-19), CDK2 (M2), CDK4 (H-22) and CDK6 (C-21) antibodies were purchased from Santa Cruz Biotechnology Inc. (Santa Cruz, CA). Polyclonal anti-OIP5 antibody (12142-1-AP) was obtained from Proteintech (Chicago, IL). Monoclonal anti-β-actin (AC-15), anti-α-tubulin (DM1A), anti-αSMA and anti-Flag were obtained from Sigma (St. Louis, MO). Rabbit cleaved poly(ADP-ribose)polymerase (PARP) polyclonal antibody, rabbit cleaved caspase-3 polyclonal antibody, and rabbit cleaved caspase-9 polyclonal antibody were purchased from Cell Signaling (Danvers, MA), while mouse anti-caspase-8 monoclonal antibody was obtained from BD Bioscience (San Jose, CA).

### Immunofluorescence and immunohistochemistry

For immunofluorescence, cells were grown on glass coverslips and transfected with GFP-tagged OIP5 expression vector or empty vector control, fixed with 4% paraformaldehyde, permeabilized in phosphate-buffered saline (PBS) containing 0.2% Triton, and blocked with 1% bovine serum albumin (BSA). The slips were then incubated with rabbit polyclonal OIP5 antibody overnight at 4°C, washed, and incubated with tetramethylrhodamine isothiocyanate isomer R (TRITC)-conjugated goat anti-rabbit immunoglobulin. After a final wash, the cells were stained with 1 mg/ml Hoechst 33258 for 15 min to visualize the nuclei, and mounted with mounting medium for fluorescence (Vector Laboratories, Burlingame, CA, USA). The cells were examined using a laser scanning LCM 510 microscope (Carl Zeiss, Jena, Germany). For the experiment regarding β-catenin nuclear translocation, HLK3 cells were transiently co-transfected with Myc-tagged OIP5 and GFP-tagged β-catenin and then processed for IF.

Immunohistochemical staining was performed on formalin-fixed, paraffin-embedded tissue sections cut to 4-μm thickness. After rehydration, a deparaffinized section was pretreated by microwave epitope retrieval (750 W for 15 min in 10 mM citrate buffer; pH 6.0). Before applying primary antibody, endogenous peroxidase activity was inhibited with 3% hydrogen peroxide, and a biotin with bovine albumin blocking step was performed. The primary antibody for OIP5 was detected using a secondary biotinylated antibody and a streptavidin-horseradish peroxidase conjugate following the manufacturer's instructions (DAKO, Glostrup, Denmark).

### shRNA expressing lentiviral vectors, transduction, and FACS analysis

A lentivirus vector encoding an shRNA targeting OIP5 (shO-1 target sequence: 5'-CCGGGCATCAGAGATGGATATTCAACTCGAGTTGAATATCCATCTCTGATGCTTTTTG-3' and shO-2 target sequence: 5'-CCGGCCATGTGTCCTCTGATCTAAACTCGAGTTTAGATCAGAGGACACATGGTTTTTG-3'), or an shRNA non-target control (NT sequence: 5'-CCGGCAACAAGATGAAGAGCACCAACTCGAGTTGGTGCTCTTCATCTTGTTGTTTT-3') were used for transduction of Huh7 and HLK2 cells according to the manufacturer's instructions (Sigma-Aldrich, St Louis, MO, USA). In brief, 5 × 10^5^ cells were incubated overnight in a 6-cm plate and transduced with lentiviral particles at a multiplicity of infection (MOI) of 1 in the presence of 8 μg/ml of polybrene. Western blot analysis was performed to confirm the knockdown of OIP5 expression. For FACS analysis, cells (2.5 × 10^5^) were cultured in a six-well plate, transduced with lentiviruses for 18 h, and treated with fresh medium. At 48 h post-transduction, cells were harvested, fixed with 70% cold ethanol, and stained with PI buffer (10 μg propidium iodide and 1 mg/ml RNAse in 1.1% sodium citrate) at 37°C for 30 min. The percentage of cells in each phase of the cell cycle was determined with a FACStar flow cytometer (Becton Dickinson, San Jose, CA, USA).

### Infection with recombinant adenovirus

The human GFP-tagged OIP5 gene cloned into the pENTR 2B vector was cloned into a pAd/CMV/V5 destination vector using the LR reaction kit (Invitrogen, Carlsbad, CA, USA). pAd/CMV/V5-OIP5 linearized with *Pac*I was transfected into 293A cells using Lipofectamine 2000 (Invitrogen). Adenoviral stock was prepared from the supernatant and was purified using an adenovirus purification kit (Clontech, Palo Alto, CA, USA). Adenoviruses encoding β-galactosidase (LacZ) served as a control.

### 3-(4,5-Dimethylthiazol-2-yl)2,5-diphenyl tetrazolium bromide (MTT) assay

Viable cells were adjusted with medium to a concentration of 2 × 10^4^ cells/ml and 1 ml per well was plated in 24-well plates. Cells were incubated for five days, then 50 μl of 3-(4,5-dimethylthiazol-2-yl)-2,5-diphenyl tetrazolium bromide (5 mg in 0.9% sodium chloride; Sigma) was added, and the cells were incubated for 4 h at 37°C. After dissolving the precipitated dye in 200 μl of dimethyl sulfoxide (Sigma) and 50 μl of glycine buffer (pH 10.5), the absorbance was read at 540 nm in a Versa Max microtiter plate reader (Molecular Devices, Sunnyvale, CA, USA).

### Soft agar colony formation assay

Cells were plated at a density of 1 × 10^5^ cells per well in 60-mm plates in growth medium containing 0.35% agar (3 ml per well) on top of a layer of growth medium containing 0.7% agar (5 ml per well). Growth medium (500 μl) with 10% FBS was added on top of the agar. The cell suspension was plated and cultured in a 37°C incubator for two weeks, after which viable colony formation was observed using an optical microscope.

### Phospho-proteome profiling

Cells were rinsed with cold PBS and immediately solubilized in NP-40 lysis buffer (1% NP-40, 20 mM Tris-HCl [pH 8.0], 137 mM NaCl, 10% glycerol, 2 mM EDTA, 1 mM sodium orthovanadate, 10 μg/ml aprotinin, 10 μg/ml leupeptin) by rocking the lysates gently at 4°C for 30 min. Following microcentrifugation at 14,000 × g for 5 min, the supernatants were transferred into a clean tube, and sample protein concentrations were resolved using the Pierce Protein Assay Kit (Pierce, Rockford, IL, USA). Lysates (500 μg) were diluted and incubated with the Human phospho-mitogen-activated protein kinase (MAPK) Array Kit (Proteome Profiler Array; R&D Systems, Minneapolis, MN, USA) per the manufacturer's instructions. Array data were developed on X-ray film following exposure to chemiluminescent reagents.

### Animal experiments

Four-week-old female BALB/c nude mice (BALB/cByJ-*Hfh11^nu^*, Orient Co., South Korea) were used in the present experiment. All mice were fed *ad libitum* and received humane care under germ-free conditions in compliance with Korean NIH guidelines. In tumorigenicity experiments, Huh7 cells were transduced with a lentivirus vector encoding an shRNA targeting OIP5. These cells were harvested and 2 × 10^6^ cells were resuspended in 60 μl DMEM with 20 μl Matrigel, and were then orthotopically injected into the left lobes of mouse livers. The mice were euthanized, and the tumor nodules and masses in the liver and mesentery were counted (n = 7).

### Luciferase reporter gene assay

Transcriptional activity assays were measured with the Luciferase Assay System (Promega, Madison, WI, USA) according to the manufacturer's instructions. HEK293T cells were simultaneously cotransfected with OIP5 or a LacZ expression plasmid, in addition to a Cdc2-luc, Cdc25C-luc, cyclin B1-luc, cyclin A-luc, cyclin D-luc, or cyclin E-luc reporter plasmid. Luciferase activity was measured using a Dual-Luciferase Reporter (DLR) Assay Kit (Promega) according to the manufacturer's instructions. Firefly and Renilla luciferase activity was gauged for normalization using a luminometer (Lumat LB9507, Berthold, Bad Wildbad, Germany). TCF/LEF-dependent transcriptional activity was assessed using TOP flash/FOP flash reporter plasmids in HEK293T cells.

### miRNA profiling and data validation

Total RNA that included small non-coding miRNA was isolated from HLK3 and Huh7 cells using an miRNEasy RNA isolation kit (Qiagen, Valencia, CA, USA) according to the manufacturer's instructions. RNA quality was determined using a Qubit 2.0 Fluorometer (Invitrogen). For each array, a minimum of 250 ng total RNA was reverse transcribed using an miScript II RT Kit (Qiagen) according to the manufacturer's instructions. For miRNA profiling studies, a SYBR green-based pathway-focused miScript miRNA PCR Array (Qiagen) was used. The Human Cancer PathwayFinder miRNA PCR Array (384-well format, MIHS-102Z) enabled simultaneous detection of 84 miRNAs previously identified in human cancers, as well as appropriate housekeeping assays and RNA quality controls. The assay was performed according to the manufacturer's protocol. Real-time fluorescence was measured in 384-well plates using the ABI Prism 7900HT Sequence Detection System (Applied Biosystems). Each condition was run in quadruplicate. Array analysis was performed using miScript miRNA PCR-array data analysis. Validation of miRNA profiling data was performed with an miScript SYBR Green PCR kit by real-time PCR estimation of miR-15b-5p. The RNU6-2 housekeeping miRNA was used for normalization.

### Transfection with miR- or inhibitor miR-15b-5p

To overexpress or inhibit miRNAs, miRNA mimics (Qiagen #MSY0000417) or inhibitor-miR (Qiagen #MIN0000417) were transfected using Hiperfect transfection reagent (Qiagen) following the manufacturer's instructions.

### miRNA target prediction

The prediction of the OIP5 3' untranslated region (3'UTR) as an miRNA binding target was performed using TargetScan 6.2 (www.targetscan.org) and miRanda (www.microrna.org). To prepare luciferase constructs containing the 3'UTR of human OIP5, we cloned the 3'UTR of OIP5 into the pGL3-Control vector. The 3'UTR regions of OIP5 mRNA (NM_007280) matching miR-15b-5p sequences were synthesized via PCR and cloned into the pGL3 control vector (Promega) downstream of the luciferase open reading frame (ORF) after digestion with XbaI. The wide type primers were: forward, 5'-CTAGTCTAGATGCTAACGCACAATCGCTTAAAAT-3' and reverse, 5'-CTAGTCTAGAAGCCAATCTTTTTCAAGAAATGAC-3'. The mutant primers were: forward, 5'-TTCAACAACAGGAAAAAAATAGTCA-3' and reverse, 5'-TGACTATTTTTTTCCTGTTGTTGAA-3'. Cells were transfected with 50 nM precursor miRNA miR-15b-5p along with the wild type or mutant OIP5-3'-UTR-luciferase constructs. 48 hours after transfection, luciferase activity was measured using a dual-luciferase assay (Promega).

### Statistical analysis

Statistical analyses were performed using Prism version 6 (Graphpad Software, San Diego, CA, USA). All experiments were performed in triplicate and data were expressed as mean ± SD. Student's t-test was used for comparison between groups, and the Kruskal-Wallis test was used for overall comparisons. Clinicopathological data were analyzed using Mann-Whitney *U* tests and chi-square tests. *P* values < 0.05 were considered significant.

## SUPPLEMENTARY MATERIALS FIGURES AND TABLES




