# Supplementary material for: OIP5, a target of miR-15b-5p, regulates hepatocellular carcinoma growth and metastasis through the AKT/mTORC1 and β-catenin signaling pathways
Source: Oncotarget. 2017 Feb 8;8(11):18129–44. doi: 10.18632/oncotarget.15185 (PMC5392313; doi:10.18632/oncotarget.15185)
Supplement: Supplementary file 1 [file oncotarget-08-18129-s001.pdf]

OIP5, a target of miR-15b-5p, regulates hepatocellular carcinoma growth and metastasis through the AKT/mTORC1 and β-catenin signaling pathways

SUPPLEMENTARY FIGURES AND TABLES

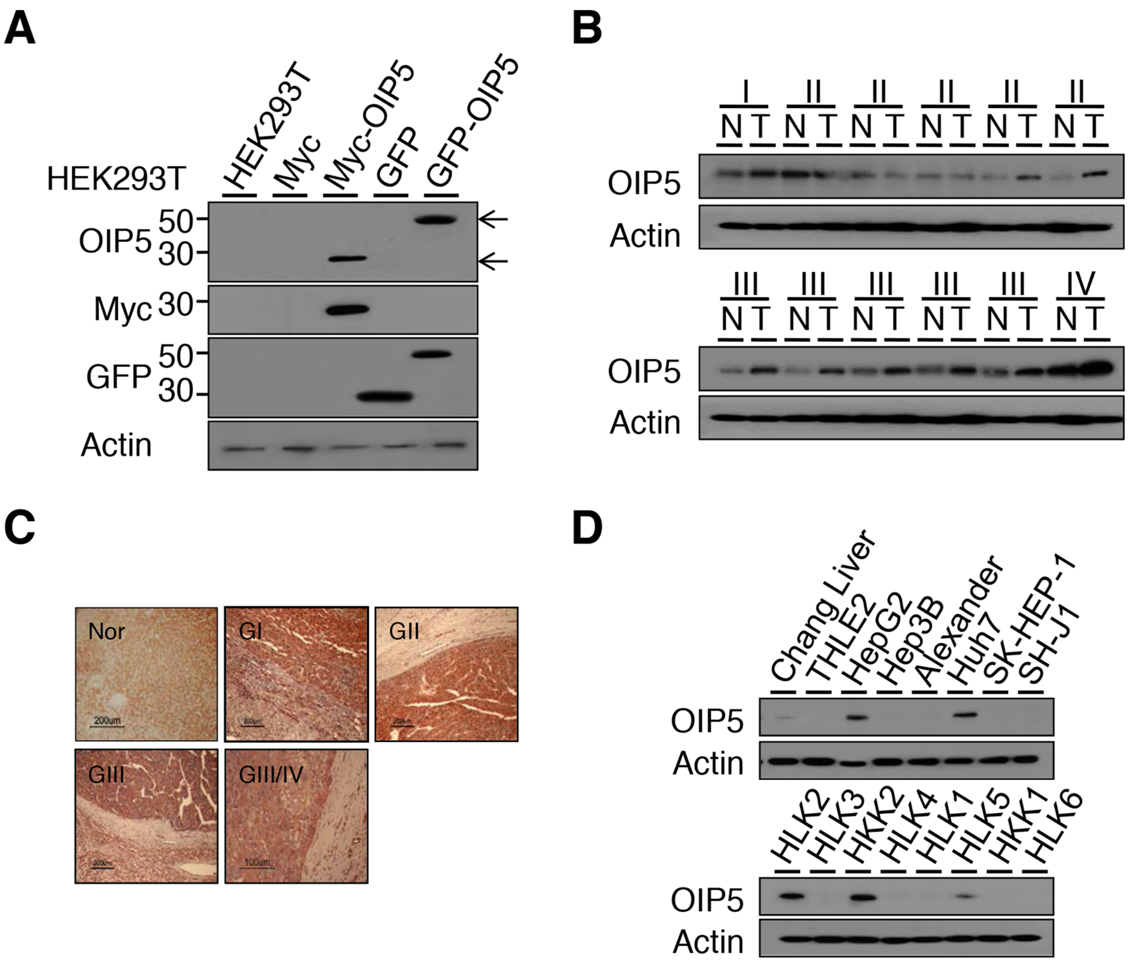

Supplementary Figure 1: OIP5 expression in HCC tissues and cell lines. A. Specific immunoreactivity for OIP5. Specific immunoreactivity of anti-OIP5 antibody against OIP5 determined by Western blot analysis. Protein lysates were prepared from HEK293T cells transfected with either GFP or c-Myc vector alone or GFP- or c-Myc-tagged OIP5. The membranes were incubated with anti-GFP, anti-Myc, or anti-OIP5 antibodies. Arrows, OIP5 proteins. The numbers on the left correspond to molecular weight. **B.** Western blot analysis of OIP5 expression in HCC tissues compared with paired corresponding non-tumor tissues (T, tumor; N, non-tumor tissues). **C.** OIP5 immunoreactivity was correlated in part with the degree of Edmondson differentiation. Normal liver; GI to GIV, Edmondson grades I to IV. D. Western blot analysis of OIP5 expression in HCC cell lines.

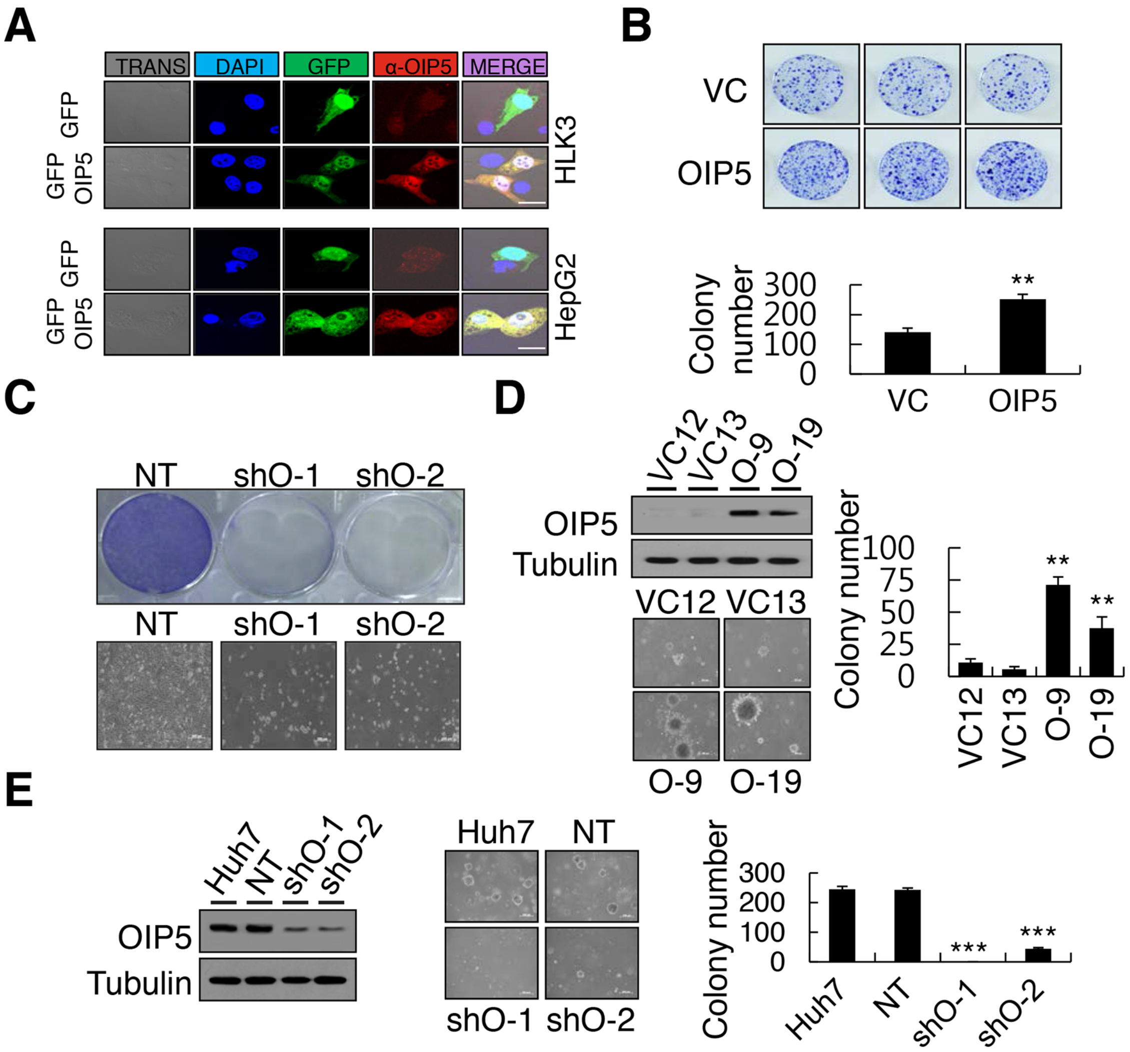

Supplementary Figure 2: OIP5-induced HCC tumor growth. A. Cellular localization of OIP5. HLK3 and HepG2 cells were transfected with the GFP-tagged OIP5 expression vector (GFP-OIP5) or empty vector control (GFP), and antibody immunoreactivity (red) was assayed via immunofluorescence. Trans, transmission; Bar, 20 μm. **B.** Colony generation assay on OIP5-expressing HLK3 cells. The colonies shown are two weeks old (upper panels). Quantification of colony formation (lower panels). Each bar represents the mean ± SD (n = 3). $^{**}P < 0.01$. VC, vector control. **C.** Colony generation assay on HLK2 cells with OIP5 knockdown (shO). NT, nontarget. **D.** Soft agar colony formation assay on OIP5-expressing SH-J1 cells. OIP5 expression in SH-J1 cells stably transfected with OIP5 expression plasmid. The colonies shown are two weeks old (left panels). Scale bar: 200 μm. Quantification of colony formation (right panels). Each bar represents the mean ± SD (n = 3). $^{**}P < 0.01$. **E.** Knockdown of OIP5 in Huh7 cells transduced with lentivirus encoding OIP5 shRNA (left panels). Soft agar colony formation assay of Huh7 cells with OIP5 knockdown. The colonies shown are two weeks old (middle panels). Scale bar: 200 μm. Quantification of colony formation (right panels). Each bar represents the mean ± SD (n = 3). $^{***}P < 0.001$.

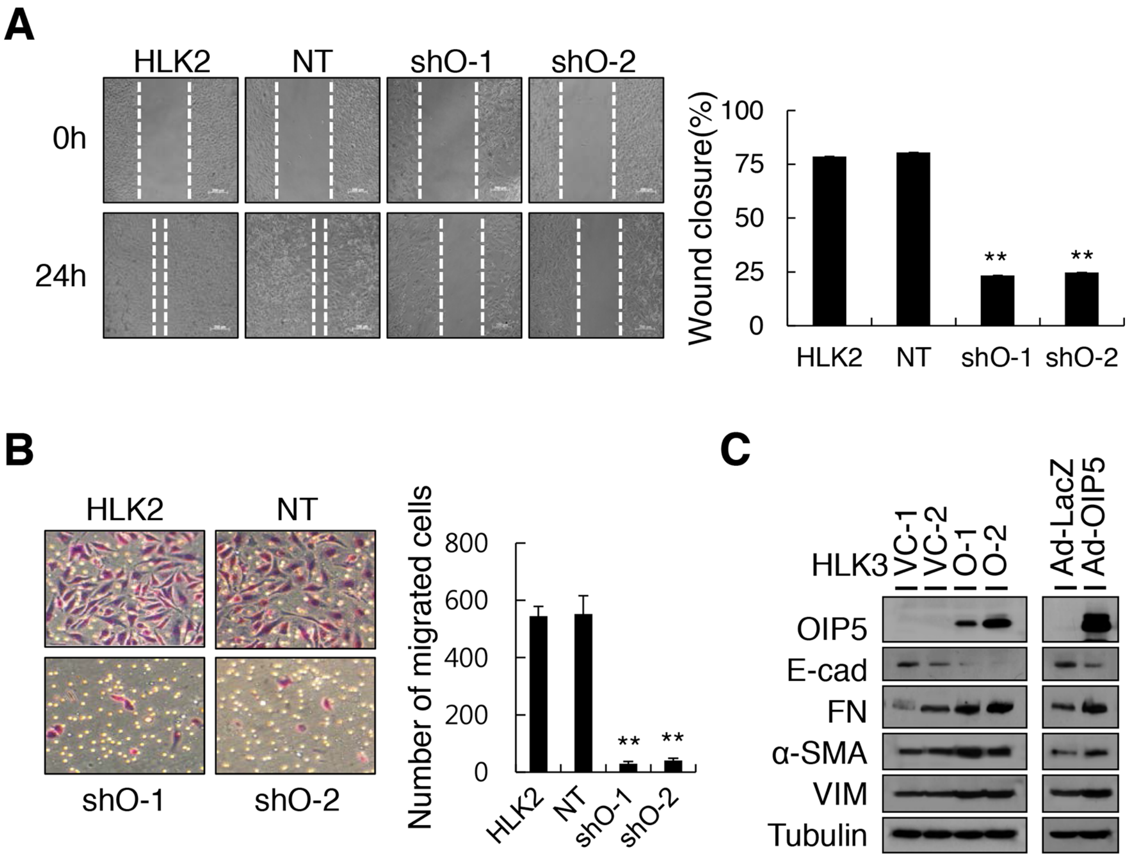

Supplementary Figure 3: Inhibition of migration and invasiveness by OIP5 knockdown. A. Migratory ability was determined in HLK2 cells transduced with lentivirus encoding OIP5 shRNA via a wound-healing assay. The results shown are representative of three independent experiments (left panels). Quantitative measurements of wound closure ability are shown (right panels) (n = 3, mean ± SD). $^{**}P < 0.01$. **B.** Modified Boyden chamber assay on HLK2 cells with OIP5 knockdown. The cells traversed the Matrigel-coated membrane after 48 h (left panels). Quantitative measurements of invasiveness are shown (right panels) (n = 3, mean ± SD). $^{**}P < 0.01$. **C.** Acquisition of mesenchymal markers and loss of epithelial marker proteins in stable OIP5 transfectants compared with vector control cells. Adeno-associated viruses expressing LacZ (control) or OIP5 were transiently transduced into HLK3 cells at a multiplicity of infection (MOI) of 100.

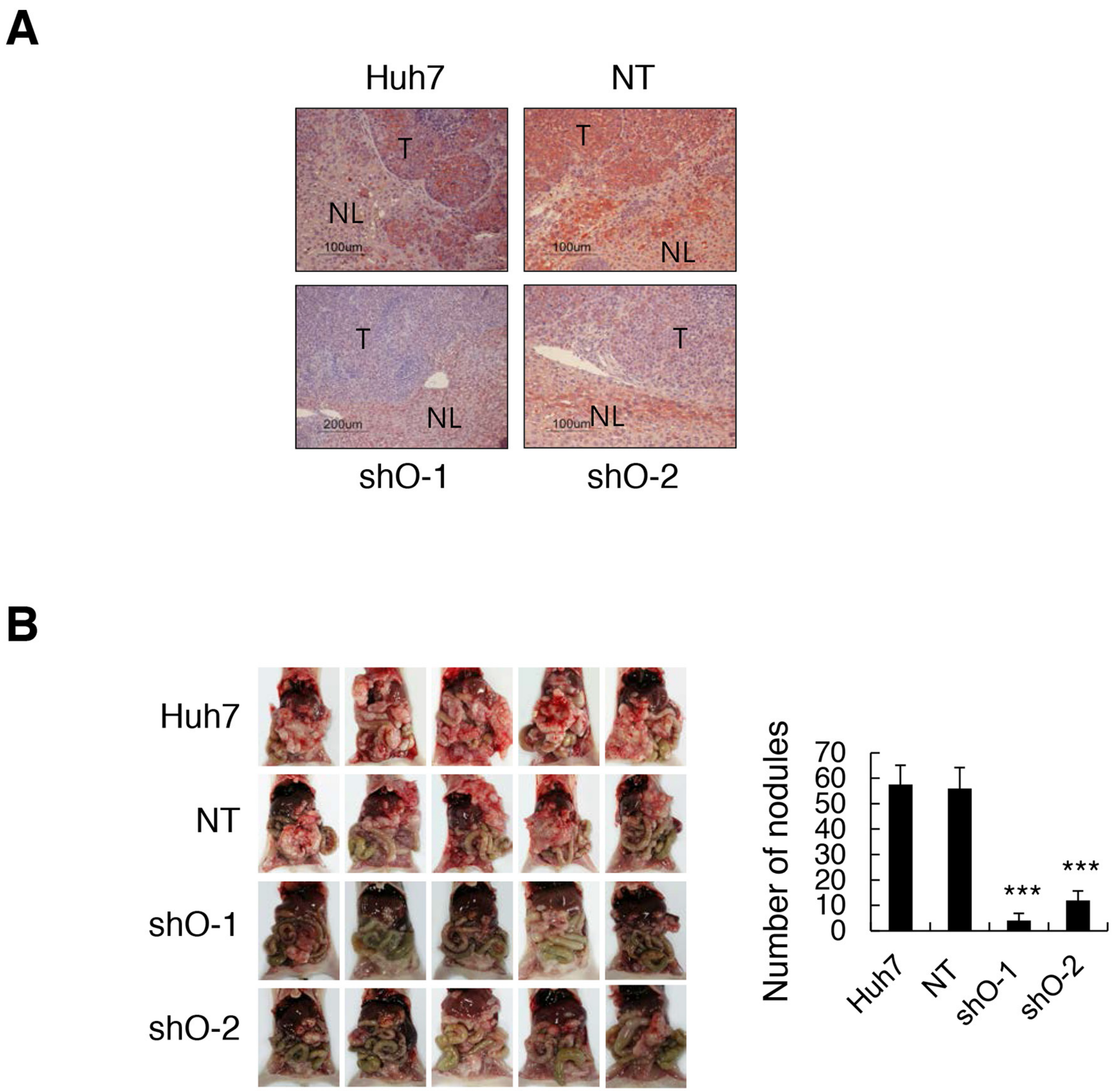

Supplementary Figure 4: OIP5 modulates tumor growth and metastatic nodule formation. A. OIP5 immunoreactivity in hepatic nodules. **B.** Metastatic nodules in the mesentery of mice (left panels). Quantification of nodules (right panels). Each bar represents the mean ± SD. $***P < 0.001$.

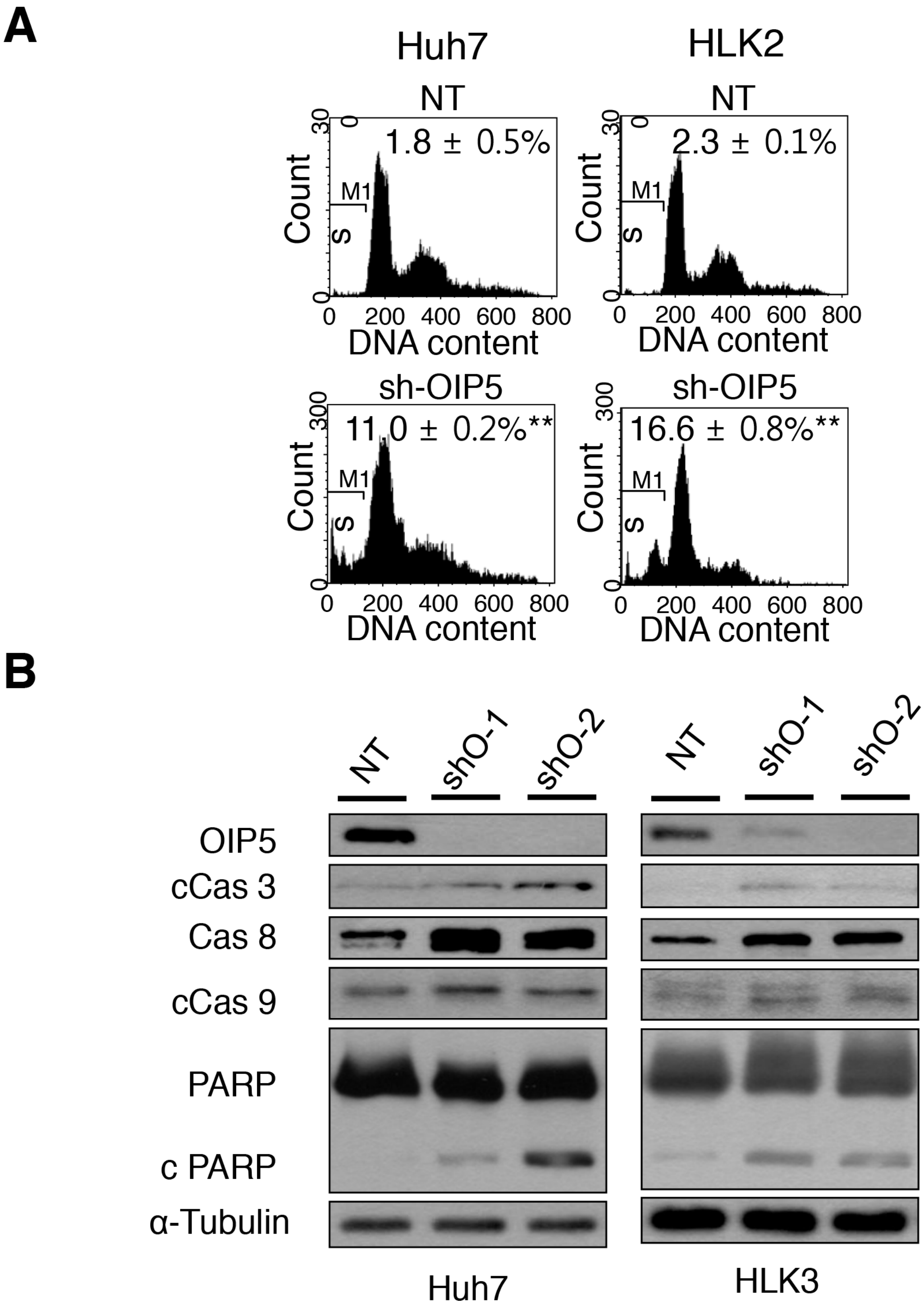

Supplementary Figure 5: Induction of apoptotic cell death by OIP5 knockdown in Huh7 and HLK2 cells. A. Quantification of the apoptotic fraction by flow cytometric analysis in Huh7 and HLK2 cells by OIP5 knockdown via lentiviral delivery of shRNA. Each value represents the mean ± SD (n = 3). ***P* < 0.01. **B.** Thirty micrograms of protein/lane isolated from cell lysates was separated using 15% SDS-PAGE. Immunodetection of PARP, caspases-3, -8, and -9 was performed by specific antibodies. Cleaved caspase, cCas; Cleaved PARP, cPARP; NT, nontarget.

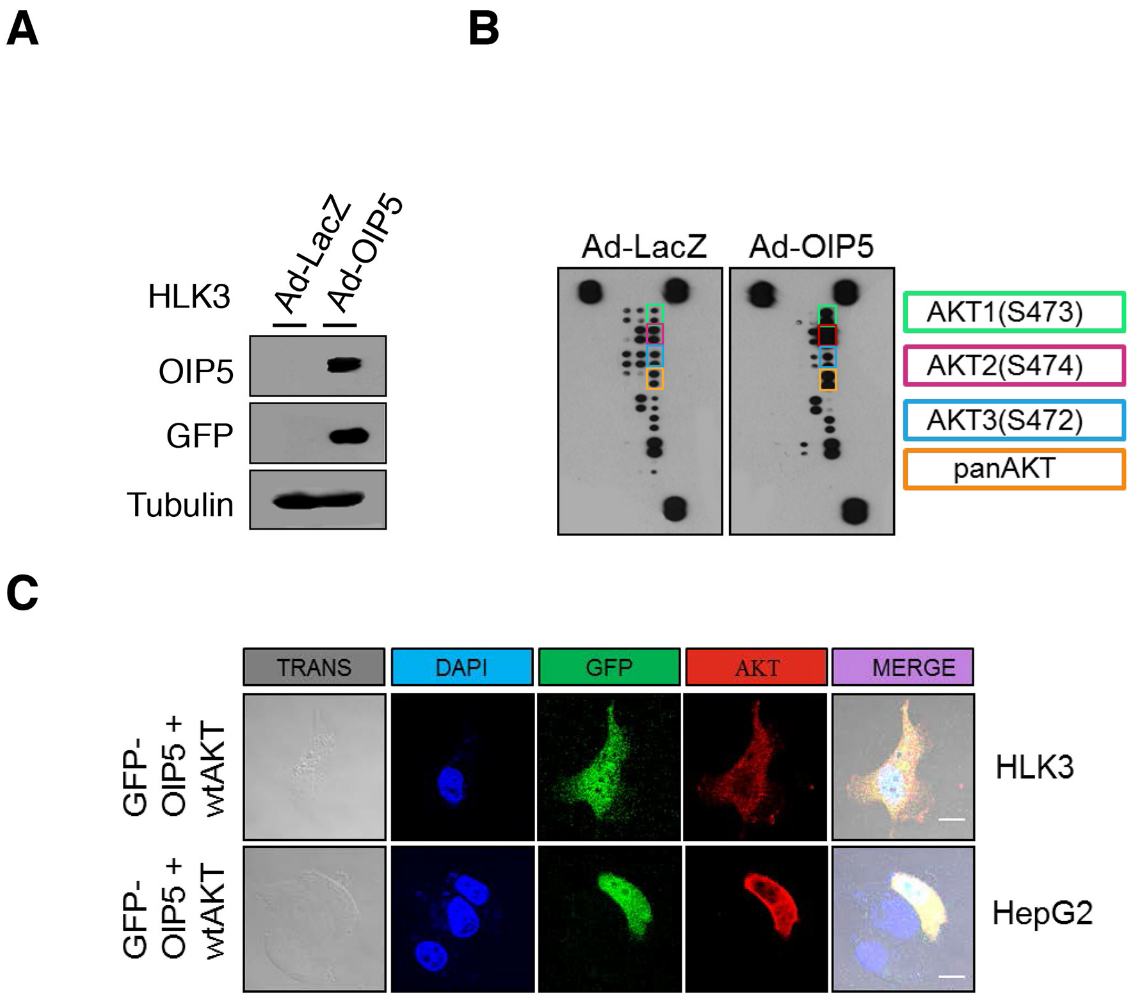

Supplementary Figure 6: Activation of OIP5 downstream molecules. A. OIP5 expression in HLK3 cells transiently infected with Ad-OIP5 and Ad-LacZ. **B.** Dot blot screens using the Human phospho-MAPK Array Kit™ based on spotted pairs (vertical doublets) of signaling protein antibodies indicated in the four boxes (AKT1, AKT2, AKT3, and panAKT). The phosphorylation state was determined using a mixture of phosphopeptide-specific antibodies. The two doublets in the corners are internal positive controls. **C.** Ectopic OIP5 was mainly localized in the nucleus, whereas wild-type AKT in the cytoplasm of the HLK3 and Hep G2 cells. Ectopic AKT was visualized via immunofluorescence assay. Scale bar, 20 µm.

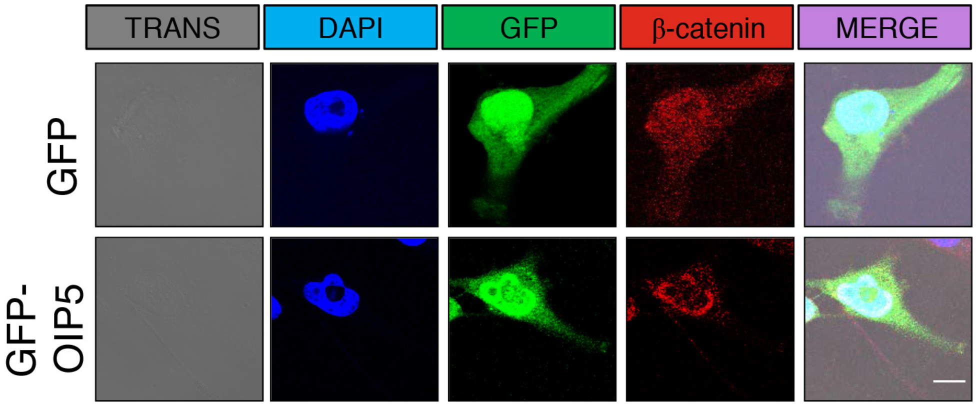

Supplementary Figure 7: OIP5 promotes the nuclear translocation of β-catenin. Confocal microscopy of HLK3 cells transiently transfected with GFP-tagged OIP5. Endogenous β-catenin was visualized via immunofluorescence assay. Scale bar, 20 μm.

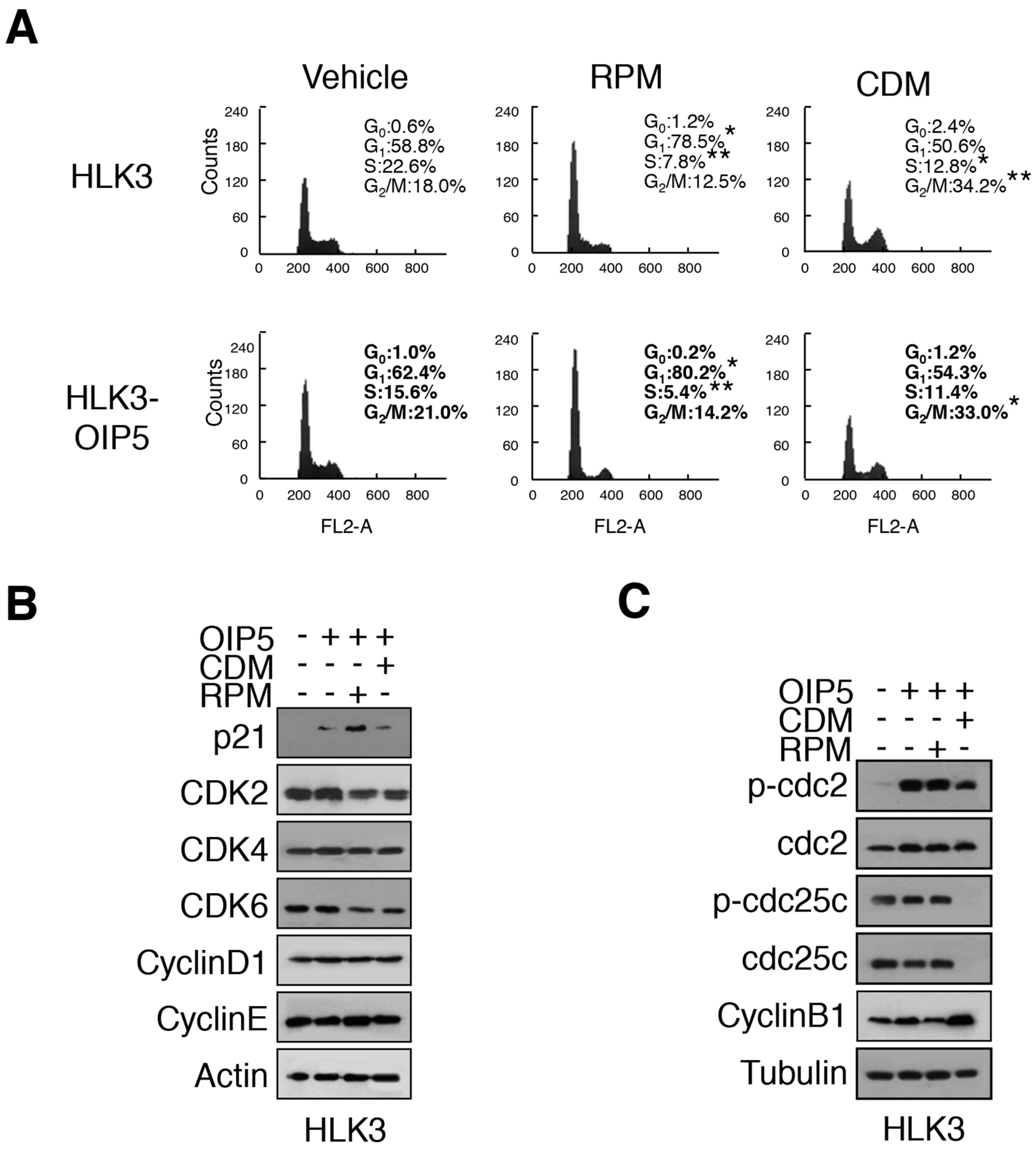

Supplementary Figure 8: Modulation of cyclins and cyclin-associated proteins. **A.** Cell cycle distribution was analyzed using flow cytometry after staining with propidium iodide (PI). HLK3 cells with or without exogenous OIP5-expression were treated with rapamycin (RPM, 100 nM) or cardamonin (CDM, 10 μM) for 24 h. $*P < 0.05$; $**P < 0.01$. n = 3. **B.** Changes in OIP5-expressing HLK3 cells in expression of G_1/S cyclins and associated proteins, including p21, CDK2, CDK4, CDK6, cyclin D1, and cyclin E, following treatment with either RPM or CDM. Immunoblots were probed with the indicated antibodies. β-Actin was used as a loading control. **C.** Changes in expression and activation in OIP5-expressing HLK3 cells of G_2/M cyclins and associated proteins, including Cdc2, Cdc25C, and cyclin B1, following treatment with either RPM or CDM. Immunoblots were probed with the indicated antibodies. Tubulin was used as a loading control.

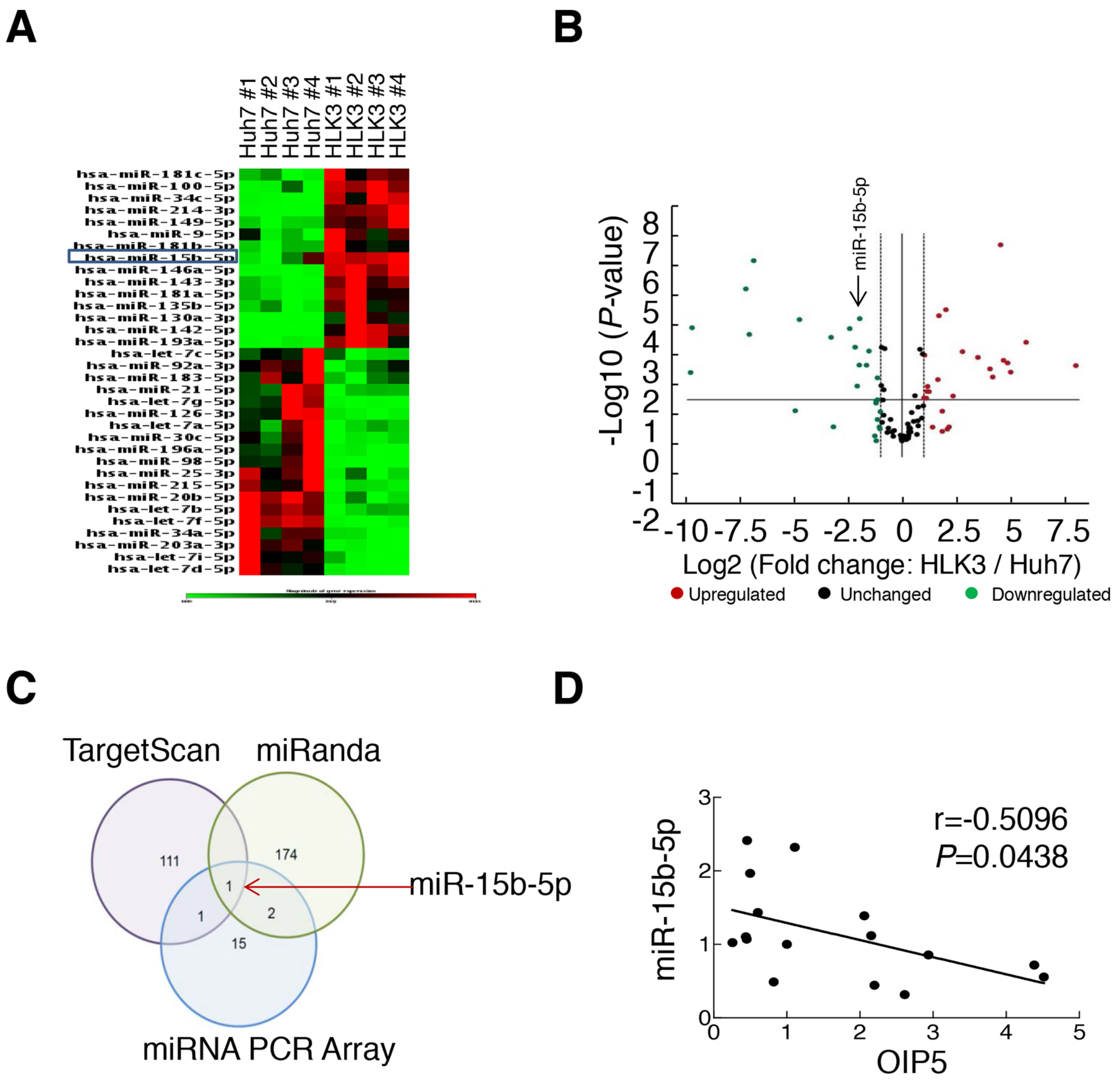

Supplementary Figure 9: Identification of miRNA 15b-5p against OIP5. A. Hierarchical clustering plot (heatmap) of miRNA profiling in HCC cell line Huh7 cells compared with immortalized cell line HLK3 cells. Red: maximum fold difference in expression (higher expression); green: minimum fold difference in expression (lower or same expression). **B.** Volcano plot of differential miRNA expression in HLK3 and Huh7 cells. The red circles indicate upregulated miRNAs and the green circles indicate downregulated miRNAs (2-fold change). The horizontal line represents $P < 0.05$. **C.** Screening for negative regulatory miRNAs of OIP5 in HCC using an miRNA PCR array. Bioinformatic prediction of potential miRNAs targeting OIP5 by TargetScan 6.2 and the miRanda database. A combination of the three methods indicated that only miR-15b-5p potentially downregulates OIP5 in HCC. **D.** OIP5 mRNA expression significantly negatively correlated with miR-15b-5p expression in 3 immortalized liver cell lines and 13 HCC cell lines on real-time PCR.

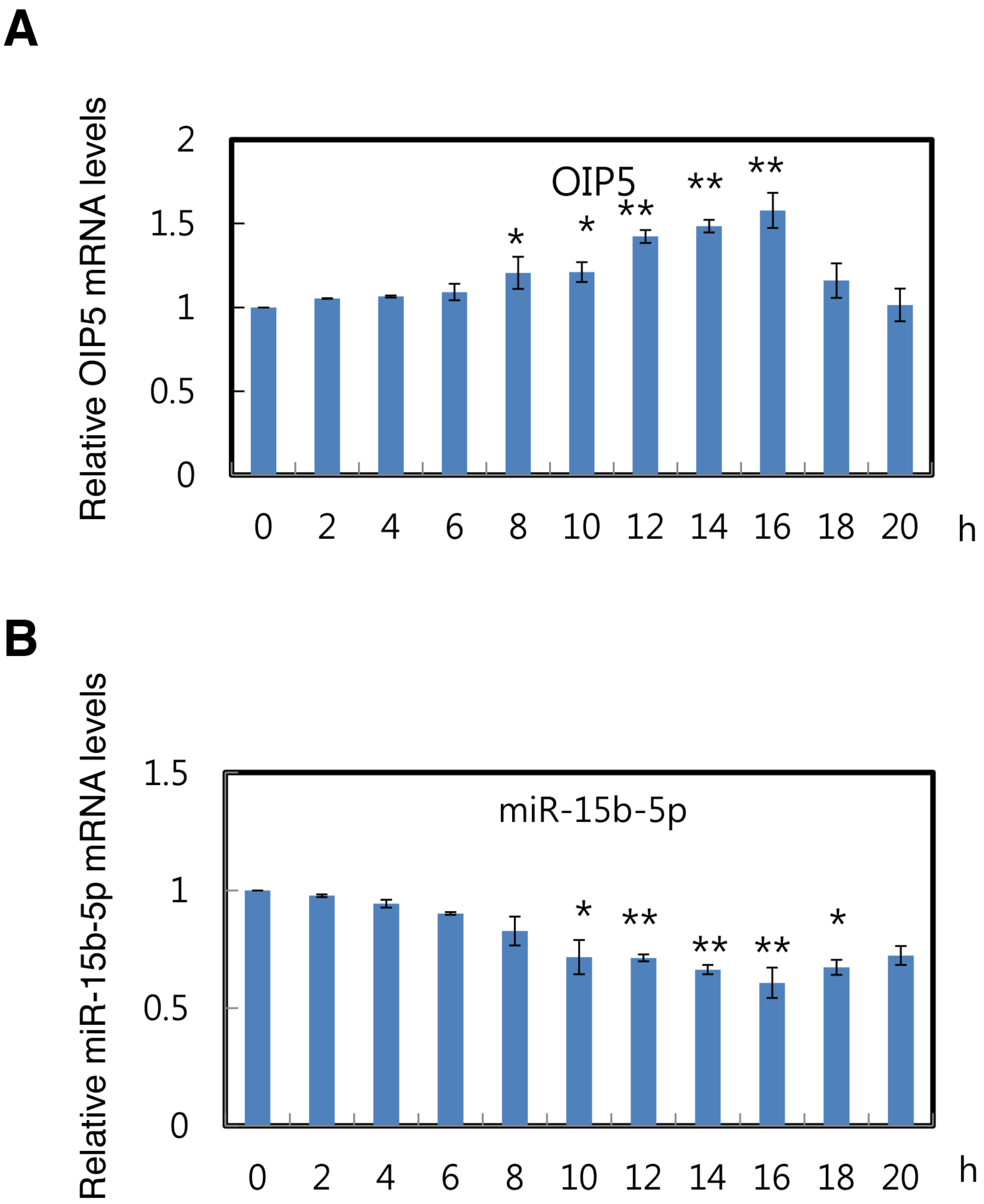

Supplementary Figure 10: Inverse relationship between OIP5 and miR-15b-5p during cell cycle progression. A. HeLa cells were synchronized in G_1 using a thymidine/aphidicolin double-block and then released. Real-time RT-PCR was used to assess the expression of OIP5 mRNA at the indicated time. Each bar represents the mean ± SD. *$P < 0.05$, **$P < 0.01$. **B.** Real-time RT-PCR was used to assess the expression of miR-15b-5p mRNA at the indicated time. Each bar represents the mean ± SD. *$P < 0.05$, **$P < 0.01$.

Supplementary Table 1: Correlation of OIP5 expression determined by real-time RT-PCR analysis with various clinicopathological features of HCC specimens

		Total	Median	Mean	95% CI of mean	*P*-value
Age (median, years)	≤58	69	22.3	29.1	22.6−35.6	0.8411
	>58	63	20.5	26.0	21.3−30.6	
Sex	Male	115	21.0	27.9	23.4−32.4	0.9783
	Female	17	23.1	25.5	16.7−34.2	
HBV	Positive	76	17.5	26.6	21.0−32.2	0.1693
	Negative	56	23.2	28.9	23.1−34.8	
Liver cirrhosis	Yes	40	14.1	23.9	17.0−30.9	0.1017
	No	92	24.0	29.2	24.2−34.1	
Tumor size (cm)	<5	101	19.4	27.1	22.2−32.0	0.2419
	≥5	31	25.3	29.1	22.4−35.9	
Edmondson grade	I	8	6.1	5.3	2.3−8.3	< 0.0001***
	II	50	13.0	19.2	14.7−23.8	
	III	68	33.0	35.8	29.5−42.1	
	IV	6	35.0	34.1	22.2−46.0	
Recurrence	Yes	58	23.0	28.8	22.3−35.4	0.8707
	No	74	21.2	26.6	21.5−31.7	
Tumor number	1	121	21.5	27.4	23.2−31.7	0.6927
	>1	11	33.3	29.2	15.3−43.2	
Fibrous capsule formation	Yes	99	17.4	25.5	21.0−30.0	0.0245*
	No	33	34.1	33.9	25.2−42.5	
Capsular infiltration	Yes	71	19.4	28.0	22.1−33.8	0.9854
	No	61	21.8	27.1	21.5−32.7	
Microvessel invasion	Yes	70	23.6	31.4	25.1−37.7	0.0481*
	No	62	18.0	23.3	18.6−27.9	
Intrahepatic metastasis	Yes	32	43.7	40.2	33.9−46.5	< 0.0001***
	No	100	15.1	23.6	18.9−28.2	
Stage (AJCC)	I/II	99	15.3	24.1	19.4−28.7	< 0.0001***
	III/IV	33	40.2	38.1	31.3−45.0	
AFP	<200	93	19.4	26.9	21.8−32.0	0.2228
	≥200	37	24.4	30.1	23.7−36.5	
	nd	2				

Abbreviations: HBV, hepatitis B virus; AFP, serum alpha-fetoprotein. Differences between subgroups were tested by the Mann-Whitney or Kruskal-Wallis tests. $^*P < 0.05$; $^{***}P < 0.001$; nd, not determined.

Supplementary Table 2: miRNAs expressed in HLK3 vs. Huh7 cells, measured by miRNA PCR array

See Supplementary File 1
