# Supplementary material for: OIP5, a target of miR-15b-5p, regulates hepatocellular carcinoma growth and metastasis through the AKT/mTORC1 and β-catenin signaling pathways
Source: Oncotarget. 2017 Feb 8;8(11):18129–44. doi: 10.18632/oncotarget.15185 (PMC5392313; doi:10.18632/oncotarget.15185)
Supplement: Supplementary file 2 [file oncotarget-08-18129-s002.docx]

**Supplementary Table 2: miRNAs expressed in HLK3 vs. Huh7 cells, measured by miRNA PCR array**

| **Mature ID** | **Fold change** | ***P*-value** |
| --- | --- | --- |
| hsa-let-7a-5p | 2.1766 | 0.045034 |
| hsa-miR-133b | 0.7516 | 0.696075 |
| hsa-miR-122-5p | 3.637 | 0.114472 |
| hsa-miR-20b-5p | 3.2645 | 0.000119 |
| hsa-miR-335-5p | 0.5101 | 0.018089 |
| hsa-miR-196a-5p | 18.4455 | 0.009753 |
| hsa-miR-125a-5p | 0.4525 | 0.050144 |
| hsa-miR-142-5p | 0.0012 | 0.000283 |
| hsa-miR-96-5p | 1.6526 | 0.227338 |
| hsa-miR-222-3p | 0.5401 | 0.051084 |
| hsa-miR-148b-3p | 1.6773 | 0.327379 |
| hsa-miR-92a-3p | 2.2692 | 0.01916 |
| hsa-miR-184 | 0.7241 | 0.509916 |
| hsa-miR-214-3p | 0.0065 | 0.000017 |
| hsa-miR-15a-5p | 0.4344 | 0.053347 |
| hsa-miR-378a-3p | 0.4955 | 0.118726 |
| hsa-let-7b-5p | 4.083 | 0.000077 |
| hsa-miR-205-5p | 1.1392 | 0.883096 |
| hsa-miR-181a-5p | 0.22 | 0.001142 |
| hsa-miR-130a-3p | 0.0011 | 0.007064 |
| hsa-miR-140-5p | 0.5532 | 0.024469 |
| hsa-miR-20a-5p | 1.7743 | 0.001332 |
| hsa-miR-146b-5p | 0.772 | 0.457363 |
| hsa-miR-132-3p | 1.2368 | 0.507468 |
| hsa-miR-193b-3p | 0.9941 | 0.742574 |
| hsa-miR-183-5p | 2.3717 | 0.027924 |
| hsa-miR-34c-5p | 0.0073 | 0.000454 |
| hsa-miR-30c-5p | 3.1586 | 0.011764 |
| hsa-miR-148a-3p | 0.4511 | 0.207001 |
| hsa-miR-134-5p | 0.524 | 0.255817 |
| hsa-let-7g-5p | 32.884 | 0.00689 |
| hsa-miR-138-5p | 0.4315 | 0.063671 |
| hsa-miR-373-3p | 0.032 | 0.110367 |
| hsa-let-7c-5p | 5.14 | 0.03851 |
| hsa-let-7e-5p | 1.3111 | 0.511041 |
| hsa-miR-218-5p | 1.2972 | 0.398855 |
| hsa-miR-29b-3p | 0.4149 | 0.677819 |
| hsa-miR-146a-5p | 0.0084 | 0.000002 |
| hsa-miR-135b-5p | 0.2515 | 0.004158 |
| hsa-miR-206 | 0.642 | 0.385062 |
| hsa-miR-124-3p | 1.2316 | 0.66337 |
| hsa-miR-21-5p | 2.2255 | 0.02761 |
| hsa-miR-181d-5p | 1.9348 | 0.001866 |
| hsa-miR-301a-3p | 0.5163 | 0.001158 |
| hsa-miR-200c-3p | 4.5165 | 0.354328 |
| hsa-miR-100-5p | 0.2552 | 0.000146 |
| hsa-miR-10b-5p | 0.619 | 0.529063 |
| hsa-miR-155-5p | 2.6606 | 0.360947 |
| hsa-miR-1-3p | 0.4801 | 0.356155 |
| hsa-miR-150-5p | 4.2826 | 0.414691 |
| hsa-let-7i-5p | 29.7482 | 0.00354 |
| hsa-miR-27b-3p | 1.4464 | 0.237266 |
| hsa-miR-7-5p | 1.3582 | 0.125946 |
| hsa-miR-127-5p | 1.8808 | 0.187683 |
| hsa-miR-29a-3p | 1.2483 | 0.380235 |
| hsa-miR-191-5p | 0.6875 | 0.20693 |
| hsa-let-7d-5p | 26.076 | 0.002949 |
| hsa-miR-9-5p | 0.2354 | 0.018701 |
| hsa-let-7f-5p | 23.7292 | 0.000001 |
| hsa-miR-10a-5p | 1.0481 | 0.673616 |
| hsa-miR-181b-5p | 0.4509 | 0.010396 |
| hsa-miR-15b-5p | 0.3175 | 0.004174 |
| hsa-miR-16-5p | 0.9838 | 0.882936 |
| hsa-miR-210-3p | 0.4884 | 0.405717 |
| hsa-miR-17-5p | 1.1354 | 0.702935 |
| hsa-miR-98-5p | 267.8174 | 0.004319 |
| hsa-miR-34a-5p | 11.4538 | 0.002391 |
| hsa-miR-25-3p | 2.0594 | 0.002033 |
| hsa-miR-144-3p | 1.6082 | 0.609306 |
| hsa-miR-128-3p | 0.9753 | 0.818309 |
| hsa-miR-143-3p | 0.0367 | 0.000157 |
| hsa-miR-215-5p | 6.9663 | 0.001574 |
| hsa-miR-19a-3p | 1.9728 | 0.078103 |
| hsa-miR-193a-5p | 0.1015 | 0.000565 |
| hsa-miR-18a-5p | 3.6349 | 0.487927 |
| hsa-miR-125b-5p | 1.1994 | 0.286191 |
| hsa-miR-126-3p | 16.904 | 0.005463 |
| hsa-miR-27a-3p | 1.5073 | 0.037689 |
| hsa-miR-372-3p | 1.2141 | 0.8017 |
| hsa-miR-149-5p | 0.1846 | 0.0003 |
| hsa-miR-23b-3p | 1.2633 | 0.409756 |
| hsa-miR-203a-3p | 53.9971 | 0.000803 |
| hsa-miR-32-5p | 0.4344 | 0.961649 |
| hsa-miR-181c-5p | 0.3433 | 0.001515 |
